# Integrative analysis of rumen microbiome and host metabolism reveals adaptive strategies of Boer goats to high-altitude environments

**DOI:** 10.3389/fmicb.2026.1897971

**Published:** 2026-08-25

**Authors:** Huifen Xu, Huidong Niu, Jiapei Liu, Awang Cuoji, Renqing Cuomu, De Ji, Ba Gui, Da Wa, Xiangtao Kang, Langda Suo, Yujiang Wu

**Affiliations:** 1 Institute of Animal Husbandry and Veterinary Medicine, Tibet Academy of Agricultural and Animal Husbandry Sciences, Lhasa, China; 2 Key Laboratory of Animal Genetics and Breeding on Tibetan Plateau, Ministry of Agriculture and Rural Affairs, Lhasa, China; 3 College of Animal Science and Technology, Henan Agricultural University, Zhengzhou, China

**Keywords:** Boer goat, fecal microbiota, high altitude, ruminal microbiota, serum metabolomics, volatile fatty acid

## Abstract

The transition to high-altitude environments presents significant metabolic challenges for livestock, primarily due to hypoxia and altered dietary resources. Understanding the adaptive strategies involving the rumen microbiome and host metabolism is crucial for improving animal health and productivity in these regions. This study investigated the adaptive responses of Boer goats to high-altitude exposure by integrating ruminal fermentation, ruminal and fecal microbiota, and serum metabolomics. The results demonstrated that high-altitude exposure markedly reshaped ruminal fermentation, microbiota composition, and serum metabolome of Boer goats. Concomitant with the elevational shift, the ruminal pH increased, whereas acetic acid concentration and the acetate-to-propionate ratio decreased; however, fecal volatile fatty acid concentrations remained unchanged. Microbial alpha diversity was reduced in both ruminal and fecal samples. The high-altitude rumen of Boer goats was characterized by an enrichment of fiber-degradation-associated taxa, accompanied by significantly elevated relative abundances of Firmicutes_A, *Ruminococcus_E*, as well as a heightened Firmicutes-to-Bacteroidetes ratio; in contrast, the relative abundances of fecal Patescibacteria, *Faecousia*, and *Phocaeicola_A* were decreased. Serum metabolomics profiling revealed that the acclimatization of Boer goats to high altitudes was characterized by profound alterations in energy and amino acid metabolism, coupled with hydromineral regulation and neuroactive metabolite pathways. Notably, energy-related metabolites, including 2-oxoglutaric acid, 3-hydroxybutyric acid, and L-malic acid, were elevated in the high-altitude cohort and positively correlated with fibrolytic taxa such as Fibrobacterota, *Fibrobacter* and *Ruminococcus_E*. In summary, the metabolic synergy between the rumen microbiome and the host likely contributes to maintaining energy homeostasis in Boer goats in high-altitude environments. These findings provide a useful reference for developing targeted nutritional strategies to improve goat farming in plateau regions.

## 1 Introduction

The Qinghai-Tibetan Plateau, widely recognized as the “Roof of the World” and the “Third Pole”, subjects livestock to severe physiological homeostasis challenges due to its unique extreme climatic conditions and distinctive topography—including hypobaric hypoxia and intense ultraviolet radiation (Jing et al., 2022). As an important economic animal in Tibet, the goat's adaptability to extreme environments directly impacts the economic income of local herders. Indigenous Tibetan goats on the Qinghai-Tibet Plateau have undergone long-term adaptive evolution, gradually developing distinct characteristics centered on cold tolerance, hypoxia tolerance, and efficient energy metabolism. They exhibit strong adaptability to the extreme high-altitude environment, and continually provide herders with high-quality meat, leather, wool, and other animal products, however, their growth rate is extremely slow (Langda et al., 2020).

The introduction of superior breeds from low-altitude regions represents an effective strategy for improving the quality of plateau livestock. Boer goats, originating from South Africa, are characterized by large body size, rapid growth and reproductive rates, superior meat quality, and broad adaptability (Guo et al., 2018). It has been successfully introduced to different regions of China, and crossbreeding with local breeds has shown significant effects in improving production performance and refining local breeds (Yao et al., 2023; Zhang et al., 2025). Boer goats have been successfully introduced to the alpine pasture regions to survive in the harsh environment. For ruminants, these challenging conditions affect not only the host, but also their commensal microbiota, especially the diversity and composition of the rumen microbiota (Li et al., 2022). The evidence suggests that the gut microbiota plays a pivotal role in modulating the plasma metabolome (Wang et al., 2022). However, elucidating how the organism maintains homeostasis through metabolic remodeling when introduced breeds are subjected to high-altitude cold and hypoxic stress remains a prominent hotspot and challenge in current research.

As the signature organ unique to ruminants, the rumen harbors a complex and diverse microbial ecosystem that is intimately associated with host nutrient absorption, immune function, and health status (Mayulu and Christiyanto, 2025). Furthermore, the gastrointestinal tract plays a pivotal role in facilitating host adaptation to high-altitude hypoxic environments by generating volatile fatty acids (VFAs) as an energy source for the host (Yang et al., 2024; Zhao et al., 2023). The diverse rumen and hindgut microbiota can effectively degrade plant fibers and convert them into energy substrates utilizable by the host, specifically VFAs, including acetate, propionate, butyrate, and valerate (Xie et al., 2023). Current research indicates that the metabolic activity of the gastrointestinal microbiota can be modulated by various factors, such as diet, breed, age, and environment. The geographical environment (including factors such as climate and altitude) and diet are key factors driving changes in the microbiota (Furman et al., 2020; Guo et al., 2023). Concurrently, serum metabolomics can systematically reflect changes in the organism's metabolic end-products under specific environmental conditions.

In recent years, multiple studies have compared the rumen microbiota of indigenous and introduced ruminants on the Qinghai-Tibetan Plateau, characterizing the tripartite interaction among the host, the environment, and the rumen microbiota. Zhang et al. (2022) found that rumen fermentation and bacteria characteristics of highland Sanhe heifers had a lower acetate-to-propionate ratio as alternative hydrogen electron acceptors for CH_4_ mitigation, a lower relative abundance of Actinobacteria, and higher relative abundance of Spirochaetae than that in rumen of lowland heifers. Huang et al. (2016; 2021) have demonstrated that rumen archaea and bacteria differ among indigenous (yak and Tibetan sheep) and introduced ruminants (cattle and crossbred sheep). Although previous studies have compared the differences between Tibetan goats and lowland goats, systematic research is currently lacking regarding how the rumen microbiota and host metabolism of low-altitude goats, following a period of adaptation to the plateau, modulate energy metabolic adaptation through structural remodeling and functional regulation. Therefore, it is imperative to unravel changes in gut microbiota and blood metabolites as well as their associations following ascent to high altitude.

To gain a deeper understanding of the dynamic changes in the gastrointestinal microbiota and serum metabolites following an increase in altitude, this study focused on goats introduced from Shaanxi to Tibet for 1 year and goats at the place of origin. By integrating microbiomics and metabolomics, this study aimed to elucidate the impacts of the high-altitude environment on the rumen microbiota structure, fermentation function, and host serum metabolic profiles of the introduced goats. The findings will provide a theoretical basis for feeding management and stress-resistance breeding of goats introduced to the plateau.

## 2 Materials and methods

### 2.1 Animals and sample collection

Boer goats raised at two distinct altitudes were selected as experimental subjects (approximately 24 months of age, half male and half female per group, mean body weight of 43.25 kg, n = 8 per group). All animals were in good health. The study was conducted at two locations: Lang County, Nyingchi City, Tibet Autonomous Region (28°40′-29°29′N, 92°28′E, altitude 3,200 m) and Linyou County, Baoji City, Shaanxi Province (34°33′-34°58′N, 107°19′-108°2′E, altitude 1,271 m). The goats in Lang County, which had been introduced from Linyou County, Shaanxi, and acclimatized for 1 year, were managed under a semi-housing system and designated as the high-altitude group (group HB). In contrast, the local goat population in Linyou County was reared under a full-housing system and served as the low-altitude group (group LB). For the HB group in Lang County, sampling was conducted on June 27, 2025, with an average ambient temperature of approximately 16 °C, an estimated atmospheric pressure of 68.3 kPa, and an effective oxygen concentration of 14.1%. In contrast, sampling for the LB group in Linyou County was performed on July 25, 2025, with an average ambient temperature of 24 °C, an estimated atmospheric pressure of 87.0 kPa, and an effective oxygen concentration of 18.0%. The LB goats were fed a standardized basal diet (Table 1). The HB goats foraged on natural alpine pasture during the daytime and were individually supplemented with 0.4 kg of a concentrate diet (Table 2) upon returning to the shelter. This research was designed and conducted in accordance with the guidelines of the Institutional Animal Care and Use Committee of the College of Animal Science and Technology of Henan Agricultural University (Approval No. HNND2025041501).

**Table 1:** Composition and nutrient levels of the basal diet for (DM basis).

Ingredients	Content (%)	Nutrient levels^b^	Content (%)
Corn stover	39.00	DM	88.90
Corn	22.81	DE (MJ/kg)	9.38
Alfalfa meal	16.46	CP	12.90
Wheat bran	6.00	Ca	0.84
Cottonseed meal	5.00	P	0.52
Rapeseed meal	4.00	CF	14.30
Soybean meal	2.77	EE	1.67
CaHPO_4_	1.48	Ash	6.72
Premix^a^	1.50		
NaHCO_3_	0.50		
NaCl	0.48		

^a^The premix was: Cu 500 mg, Zn 2,500 mg, Mn 2, 500 mg, I 30 mg, Se 10 mg, Co 35 mg, VA 150,000 IU, VD 60,000 IU, VE 1,500 IU.

^b^All data was measured.

**Table 2:** Composition and nutrient levels of the concentrate diet (DM basis).

Ingredients	Content (%)	Nutrient levels^b^	Content (%)
Corn	51.55	DM	88.70
Wheat bran	11.82	DE (MJ/kg)	13.23
Wheat middlings	9.27	CP	15.12
Soybean meal	15.25	CF	5.38
Rapeseed meal	7.11	Ca	1.24
CaHPO_4_	1.42	P	0.55
CaCO_3_	0.98		
Premix^a^	1.00		
NaHCO_3_	0.50		
NaCl	1.00		

^a^The premix was: Cu 500 mg, Zn 2,500 mg, Mn 2,500 mg, I 30 mg, Se 10 mg, Co 35 mg, VA 150,000 IU, VD 60,000 IU, VE 1,500 IU.

^b^All data was measured.

On the morning of sampling, rumen content was collected from fasted Boer goats using an oral stomach tube prior to feeding. Approximately 50 ml of rumen fluid was aspirated, immediately filtered through four layers of sterile gauze, and then aliquoted into 15 ml cryogenic vials. These aliquots were flash-frozen in liquid nitrogen. Subsequently, fresh fecal samples were obtained via rectal grab using sterile, disposable gloves and placed into 2 ml cryotubes, which were also promptly snap-frozen in liquid nitrogen. All samples were then stored at −80 °C until further analysis. Both rumen fluid and fecal samples were processed in duplicate: one aliquot was reserved for 16S rRNA gene sequencing, and the other for volatile fatty acid (VFA) analysis. Blood samples (10 ml per animal) were collected from the jugular vein using sterile disposable needles and transferred into vacuum blood collection tubes. After standing at room temperature for 1 h, the blood samples were centrifuged at 3,000 rpm for 10 min to separate serum. The serum was carefully aliquoted into 2 ml cryovials and stored at −80 °C until further analysis for non-targeted metabolomic profiling.

### 2.2 Ruminal and fecal VFAs analysis

For rumen fluid, samples were initially diluted 5-fold with distilled water. A 200 μL aliquot of the diluted sample was sequentially mixed with 50 μL of 15% phosphoric acid, 10 μL of 75 μg/ml 4-methylvaleric acid (IS), and 140 μL of ether. For fecal samples, 100 mg of glass beads and 500 μl of water were added to the sample, followed by homogenization for 1 min. The homogenate was centrifuged at 12,000 rpm for 10 min at 4 °C. Then, 200 μl of the resulting supernatant was aliquoted and mixed with 100 μl of 15% phosphoric acid, 20 μl of 375 μg/ml 4-methylvaleric acid, and 280 μl of ether. Both rumen and fecal mixtures were vortexed thoroughly for 1 min using a vortex mixer (Haimen Kylin-Bell Lab Instruments Co., Ltd., China) and then centrifuged at 12,000 rpm for 10 min at 4 °C in a refrigerated centrifuge (Hunan Xiangyi Laboratory Instrument Development Co., Ltd., China). The final supernatant from each preparation was collected and transferred into autosampler vials for subsequent GC-MS analysis. Volatile fatty acids were quantified using a Trace 1310 gas chromatograph coupled with an ISQ LT mass spectrometer (Thermo Fisher Scientific, USA). The GC was performed on an Agilent HP-INNOWAX capillary column (30 m × 0.25 mm ID × 0.25 μm). High-purity helium served as the carrier gas at a constant flow rate of 1 ml/min. Samples (1 μl) were injected in split mode with a split ratio of 10:1, and the injector temperature was maintained at 250 °C. The column oven temperature program was optimized as follows: initial temperature held at 90 °C, increased to 120 °C at a rate of 10 °C/min, further elevated to 150 °C at 5 °C/min, and finally ramped to 250 °C at 25 °C/min with a terminal hold time of 2 min. The mass spectrometer was operated in electron impact (EI) ionization mode at 70 eV. The ion source and transfer line temperatures were set to 300 °C and 250 °C, respectively. Quantitative determination of the target VFAs was executed using the single ion monitoring mode.

### 2.3 Ruminal and fecal microbiome analysis

Total genomic DNA was extracted from the goat rumen fluid and fecal samples using the OMEGA Soil DNA Kit (M5635-02; Omega Bio-Tek, Norcross, GA, USA) in strict accordance with the manufacturer's instructions. The concentration and purity of the DNA were determined using a NanoDrop NC2000 spectrophotometer (Thermo Fisher Scientific, Waltham, MA, USA), and DNA integrity was verified via agarose gel electrophoresis. The V3–V4 hypervariable regions of the bacterial 16S rRNA gene from both rumen fluid and fecal samples were amplified by PCR using the specific primers 338F 5′-ACTCCTACGGGAGGCAGCA3′ and 806R 5′-GGACTACHVGGGTWTCTAAT3′. The resulting PCR amplicons were purified using Vazyme VAHTS™ DNA Clean Beads (Vazyme, Nanjing, China) and quantified using the Quant-iT PicoGreen dsDNA Assay Kit (Invitrogen, Carlsbad, CA, USA). Purified amplicons were pooled in equimolar concentrations, and paired-end sequencing was conducted on the Illumina platform at Shanghai Personal Biotechnology Co., Ltd. (Shanghai, China).

### 2.4 Microbiome data analysis

Microbiome data processing was performed using QIIME2 (version 2022.11) according to official tutorials. Raw sequence reads were demultiplexed via the demux plugin, and primer sequences were trimmed using the cutadapt plugin. Quality filtering, denoising, paired-end read merging, and chimera elimination were executed using the DADA2 plugin (version V1.1). Non-singleton amplicon sequence variants (ASVs) were aligned using MAFFT (RRID: SCR_011811) and utilized to construct a phylogenetic tree with FastTree2. Taxonomic assignment of ASVs was performed using the classify-sklearn naïve Bayes taxonomy classifier within the feature-classifier plugin against the Greengenes2 database (version gg2_2022_10). Alpha-diversity metrics (including Chao1, Observed species, Shannon, Simpson, Pielou's evenness) and beta-diversity metrics (Bray-Curtis distances) were calculated using the diversity plugin, with all samples rarefied to an even depth based on the minimum sequence count per sample. Statistical analyses and data visualizations were primarily conducted using QIIME2 and R packages. Alpha-diversity indices were visualized as box plots. Beta-diversity structural variations were visualized via Principal Coordinate Analysis (PCoA) and Unweighted Pair-Group Method with Arithmetic Means (UPGMA) hierarchical clustering. Significant differences in microbial community structures between groups were assessed using PERMANOVA within QIIME2. Taxonomic compositions were visualized via MEGAN (RRID: SCR_011942) and GraPhlAn (RRID: SCR_016130). Shared and unique ASVs were characterized using the R package VennDiagram (RRID: SCR_002414). Differentially abundant taxa at the ASV level were identified using Linear Discriminant Analysis Effect Size (LEfSe).

### 2.5 LC-MS analysis

Serum metabolome data were generated and analyzed using a liquid chromatography-mass spectrometry (LC-MS) platform. Briefly, a 50 μl aliquot of each serum sample was transferred into a 2 ml centrifuge tube, mixed with 200 μl of pre-cooled methanol: acetonitrile (1:1, v/v), and vortexed for 30 s. The mixture was incubated at −20 °C for 30 min to precipitate proteins and then centrifuged at 12,000 rpm for 10 min at 4 °C. A 200 μl aliquot of the resulting supernatant was collected and evaporated to dryness under vacuum. The dried residue was reconstituted in 100 μl of 50% methanol containing 5 ppm of 2-chlorophenylalanine as an internal standard, followed by vortexing for 30 s. After a final centrifugation at 12,000 rpm for 10 min at 4 °C, the supernatant was filtered through a 0.22 μm membrane and transferred into autosampler vials for LC-MS analysis. Quality control (QC) samples were prepared by pooling 10–20 μl aliquots from each filtered sample to monitor instrument stability and data reproducibility throughout the analytical run. Chromatographic separation was performed on an ACQUITY UPLC HSS T3 capillary column (100 Å, 1.8 μm, 2.1 mm × 100 mm) maintained at 40 °C. The mobile phase flow rate was set at 0.4 ml/min, the autosampler temperature was kept at 8 °C, and the injection volume was 2 μl. Both positive and negative ionization modes utilized mobile phase A (0.1% formic acid in water) and mobile phase B (acetonitrile containing 0.1% formic acid). Mass spectrometry data were acquired in both positive and negative electrospray ionization (ESI) modes using a Thermo Orbitrap Exploris 120 mass spectrometer controlled by Xcalibur software (version 4.7; Thermo Fisher Scientific; RRID: SCR_014593). The raw MS data files were imported into Compound Discoverer™ 3.3 software (version 3.3.2.31; Thermo Fisher Scientific, Waltham, MA, USA) for peak detection, alignment, and correction based on its peak-rating and quality scoring algorithms to minimize background noise and low-quality features. Features missing in more than 50% of the QC samples were filtered out, and remaining missing values were imputed using the Fill Gaps algorithm. The total peak area was normalized by sum normalization.

### 2.6 Serum metabolome data analysis

Multivariate statistical and pathway enrichment analyses to evaluate the metabolic variations among groups, Principal Component Analysis (PCA) and Orthogonal Partial Least Squares Discriminant Analysis (OPLS-DA) were performed using the ropls package in R. A distance matrix was calculated using the normalized dataset, and hierarchical cluster analysis was conducted to group all samples. Significantly differential metabolites were selected based on a strict criterion of a variable importance in projection (VIP) score >1 derived from the OPLS-DA model and *P*-value < 0.05. Metabolic pathway analysis were analyzed using MetaboAnalystR 3.0 (https://www.metaboanalyst.ca/). The statistical significance of metabolite enrichment within each pathway was determined using Fisher's exact test.

### 2.7 Statistical analysis

Statistical analysis was performed with Student's t-test to compare ruminal fermentation parameters and differential fecal VFA contents using SPSS 24.0. Values were presented as the means ± standard error of the mean (SEM). LEfSe was used to compare the relative abundance of microorganisms. Linear discriminant analysis (LDA) score >2 and *P*-value < 0.05 were considered statistically significant. A VIP > 1 and *P*-value < 0.05 were the thresholds to determine differentially expressed metabolites. Statistics and plotting were done using GraphPad Prism 8 software. Heatmap were generated using platform (https://www.genescloud.cn). Spearman correlation analysis was performed using R software (version 4.4.2) with the ggplot2, ggpubr, and psych packages. ^*^indicated a significant difference, *P* < 0.05; ^**^indicated a highly significant difference, *P* < 0.01.

## 3 Results

### 3.1 Analysis of rumen fermentation parameters

As shown in Table 3, the ruminal *p*H in the high-altitude Boer goats (HB group, pH = 6.51) was significantly higher than that in the low-altitude Boer goats (LB group, *p*H = 6.26, *P* < 0.05). In the rumen fluid, the acetic acid concentration was significantly lower in the HB group than in the LB group (*P* < 0.05), whereas the concentrations of valeric acid and isovaleric acid were significantly higher (*P* < 0.05). Additionally, the acetate-to-propionate (A/P) ratio was extremely significantly lower than that in the LB group (*P* < 0.001).

**Table 3:** Comparison of ruminal fermentation characteristics in Boer goats (μg/ml).

Items	Group		*P*-value
	HB	LB	
Acetic acid	325.23 ± 47.31	539.47 ± 41.37	< 0.01
Propionic acid	133.53 ± 22.68	109.52 ± 10.12	0.36
Isobutyric acid	6.52 ± 0.66	6.15 ± 0.61	0.69
Butyric acid	93.23 ± 13.73	83.12 ± 8.91	0.55
Isovaleric acid	6.46 ± 0.78	4.30 ± 0.48	0.03
Valeric acid	14.28 ± 2.52	5.21 ± 0.74	< 0.01
Caproic acid	0.55 ± 0.11	0.18 ± 0.03	0.01
TVFA	579.81 ± 86.55	747.94 ± 59.76	0.13
A/P	2.62 ± 0.22	4.98 ± 0.13	< 0.01
pH	6.51 ± 0.09	6.26 ± 0.05	0.04

### 3.2 Analysis of fecal volatile fatty acids

As detailed in Table 4, the fecal concentrations of acetic acid, propionic acid, total volatile fatty acids, valeric acid, and isobutyric acid in the feces of high-altitude Boer goats (HB) were lower than those in low-altitude Boer goats (LB). However, the levels of butyric acid and the acetic-to-propionic acid ratio were higher in the HB group than in the LB group, though the differences were not significant (*P* > 0.05).

**Table 4:** Comparison of volatile fatty acids in the feces of Boer goats (μg/ml).

Items	Group		*P*-value
	HB	LB	
Propionic acid	52.28 ± 6.99	57.25 ± 4.67	0.56
Acetic acid	225.57 ± 42.63	234.54 ± 17.24	0.85
Butyric acid	29.73 ± 6.67	26.55 ± 4.15	0.69
Isovalericacid	4.20 ± 0.95	4.00 ± 0.80	0.88
Valeric acid	5.01 ± 0.88	5.74 ± 0.60	0.50
Caproic acid	0.05 ± 0.01	0.05 ± 0.01	0.80
Isobutyric acid	5.99 ± 0.97	7.55 ± 1.16	0.32
TVFA	322.81 ± 52.10	335.68 ± 23.93	0.83
A/P	4.25 ± 0.34	4.14 ± 0.17	0.78

### 3.3 Ruminal microbiota diversity

**Figure 1:**
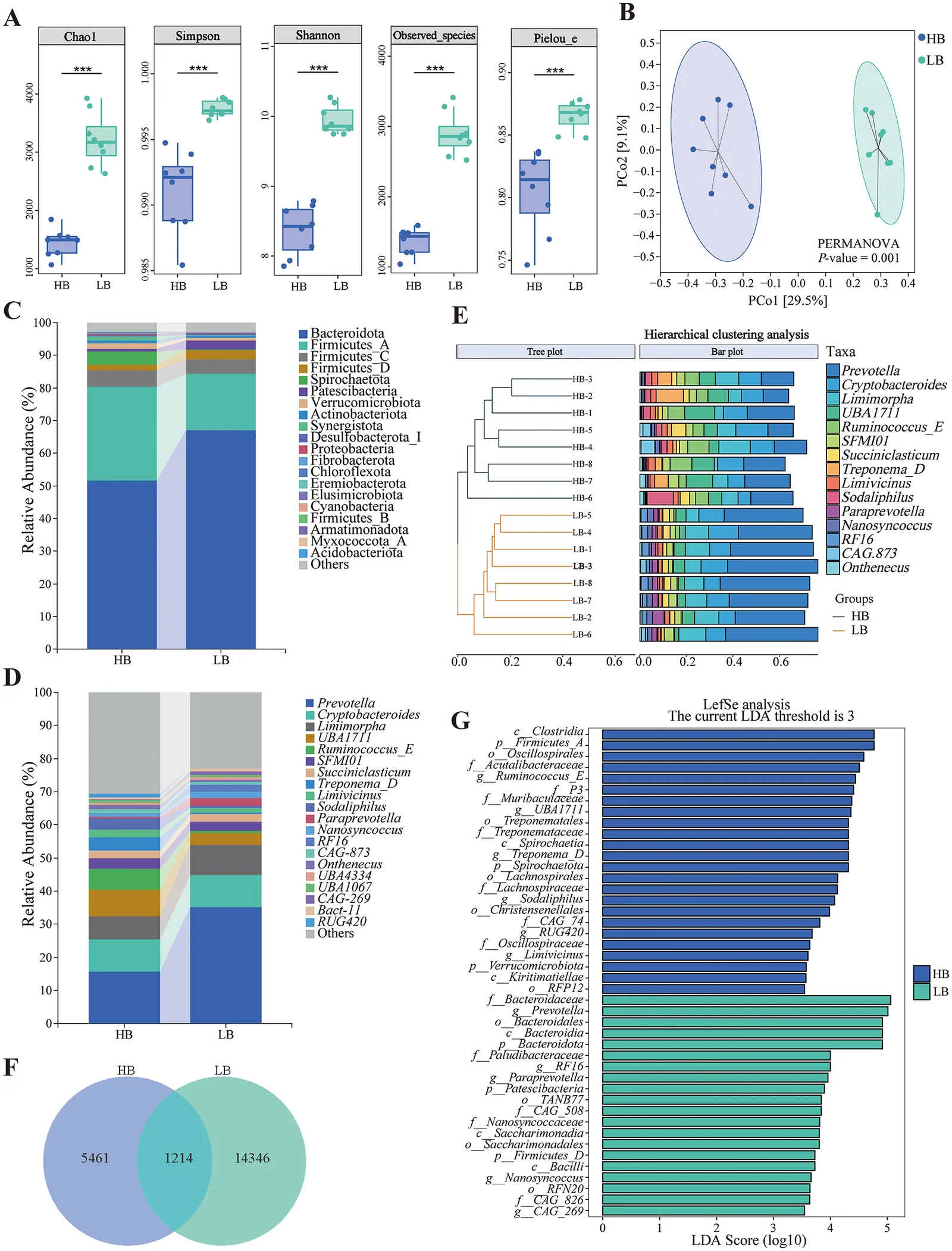
Comparison of ruminal microbiota in Boer goat at different altitudes. **(A)** Alpha diversity indices; **(B)** PCoA based on Bray–Curtis distance; **(C)** Relative abundance of the top 20 bacterial phylum; **(D)** Relative abundance of the top 20 bacterial genus; **(E)** Hierarchical clustering analysis; **(F)** Venn diagram illustrated the number of shared and unique ASVs in ruminal bacteria; **(G)** LEfSe analysis identifying differentially taxa.

As shown in Figure 1A, the alpha diversity indices, including the Chao1, Shannon, Simpson, Observed species, and Pielou_e indices, were markedly diminished in the HB group compared to the LB group (*P* < 0.01). PCoA based on the Bray-Curtis distance matrix at the ASV level was employed to evaluate the overall microbiota composition (Figure 1B), revealing a clear structural separation, with the HB and LB samples forming two distinct clusters. In addition, the PERMANOVA analysis confirmed a significant difference between the groups (*P* = 0.001).

**Figure 2:**
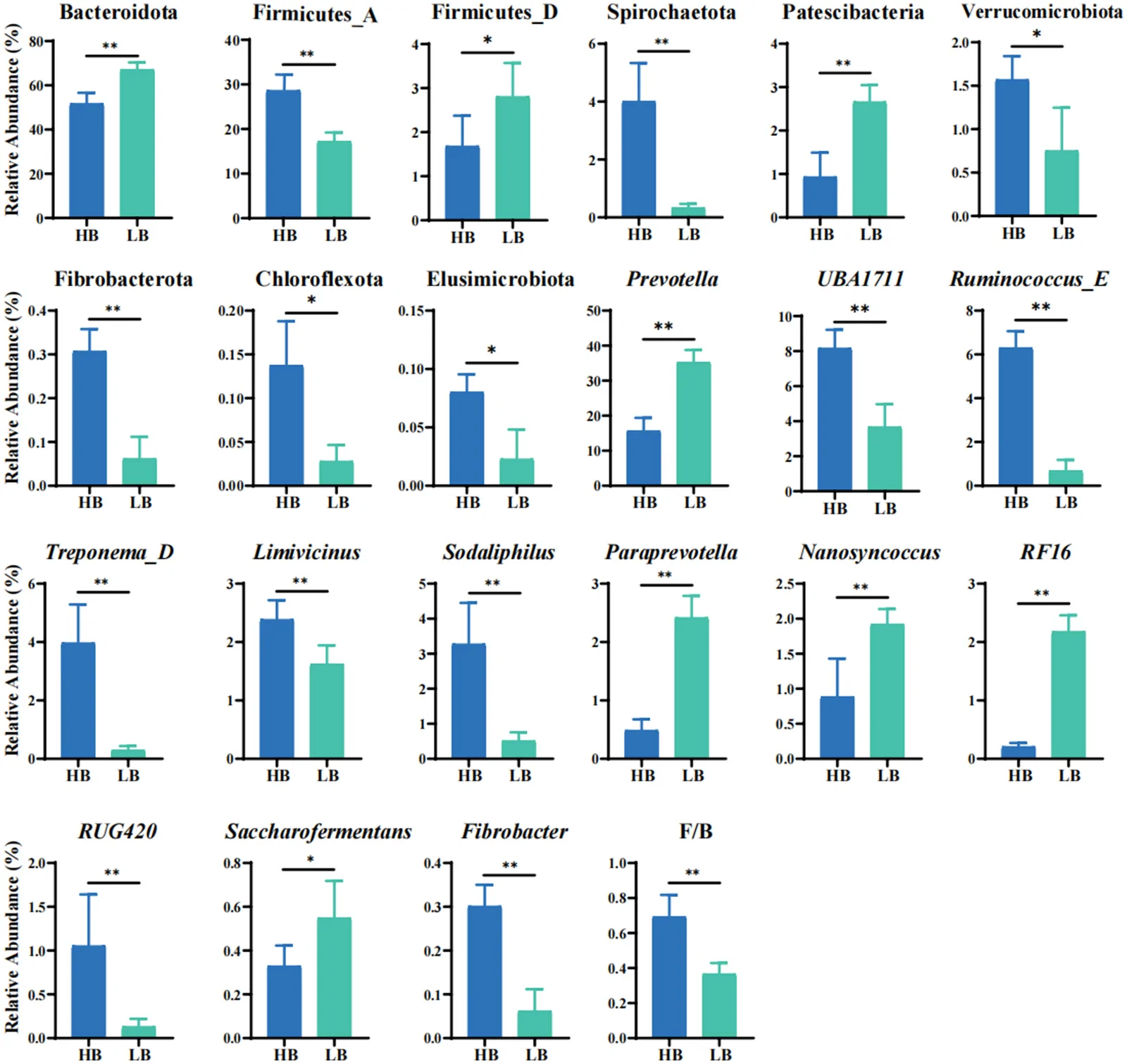
Key differentially microbial taxa in the rumen of Boer goats at different altitudes. ^*^ indicates a significant difference at P < 0.05; ^**^ indicates an extremely significant difference at P < 0.01.

At the phylum level, although Firmicutes and Bacteroidota constituted the dominant phyla in both cohorts, their relative proportions exhibited substantial variations between the two groups (Figure 1C). Furthermore, analysis of the top 20 most abundant genera (Figure 1D) identified *Prevotella, Cryptobacteroides*, and *Limimorpha* as the pre-dominant taxa across the samples. Hierarchical clustering utilizing the UPGMA algorithm based on Bray-Curtis distances consistently segregated the HB and LB samples into two separate clades (Figure 1E), underscoring the profound differences in microbial community composition between the two groups. A total of 5,461 and 14,346 unique ASVs were exclusively detected in the HB and LB groups, respectively (Figure 1F). To identify the specific microbial taxa driving these compositional shifts, the rumen microbiota was subjected to the Mann–Whitney *U*-test (Figure 2) and LEfSe analysis (Figure 1G). The analytical results demonstrated that the LB group was pre-dominantly enriched with specific lineages, including Bacteroidota, *Prevotella, Paraprevotella, Nanosyncoccus*, and *RF16* (*P* < 0.05). Conversely, microbial taxa significantly overrepresented in the HB group primarily comprised Firmicutes_A, Spirochaetota, Onthenecus, *Treponema_D, Ruminococcus_E*, and *Limivicinus*. Additionally, the Firmicutes-to-Bacteroidota (F/B) ratio was significantly higher in the HB group (*P* < 0.05).

### 3.4 Fecal microbiota diversity

**Figure 3:**
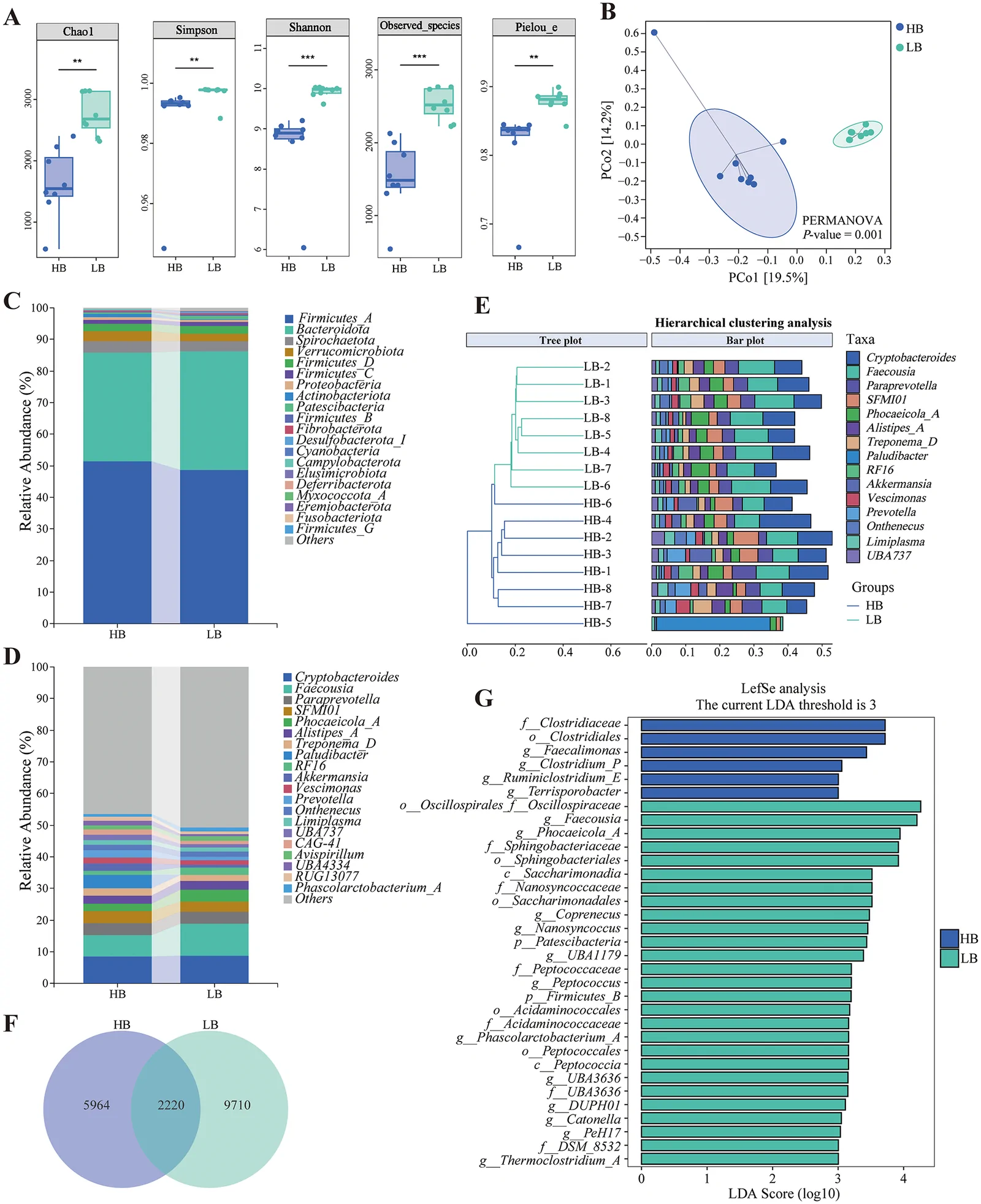
Comparison of fecal microbiota in Boer goat at different altitudes. **(A)** Alpha diversity indices; **(B)** Principal coordinate analysis (PCoA) based on Bray–Curtis distance; **(C)** Relative abundance of the top 20 bacterial phylum; **(D)** Relative abundance of the top 20 bacterial genus; **(E)** Hierarchical clustering analysis; **(F)** Venn diagram illustrated the number of shared and unique ASVs in fecal bacteria; **(G)** LefSe analysis identifying differentially taxa. ^*^ indicates a significant difference at P < 0.05; ^**^ indicates an extremely significant difference at P < 0.01; ^***^ indicates an extremely significant difference at P < 0.001.

Analysis of the fecal microbiota in Boer goats revealed that the alpha diversity indices, including the Chao 1, Shannon, Simpson, Observed species, and Pielou_e indices, were markedly diminished in the HB group compared to the LB group (*P* < 0.01, Figure 3A). PCoA based on the Bray-Curtis distance matrix (Figure 3B) demonstrated a clear structural separation, with the HB and LB samples forming two distinct clusters. Furthermore, this compositional divergence was statistically corroborated by PERMANOVA (*P* = 0.001).

**Figure 4:**
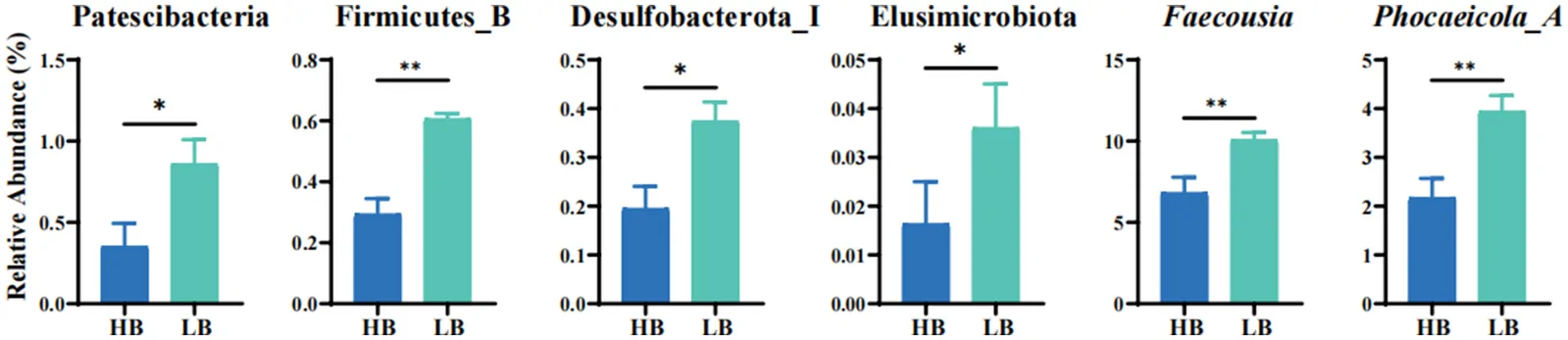
Key differentially microbial taxa in the feces of Boer goats at different altitudes. ^*^ indicates a significant difference at P < 0.05; ^**^ indicates an extremely significant difference at P < 0.01.

At the phylum level, Firmicutes_A and Bacteroidota constituted the pre-dominant phyla across fecal samples from both groups (Figure 3C). At the genus level, *Cryptobacteroides* and *Faecous* were identified as the dominant taxa across all samples (Figure 3B). Hierarchical clustering analysis based on Bray-Curtis distances consistently segregated the HB and LB samples into two separate clades (Figure 3E). Additionally, 5,964 and 9,710 unique ASVs were exclusively detected in the HB and LB groups, respectively (Figure 3F). Mann–Whitney *U*-test (Figure 4) and LEfSe (Figure 3G) showed that the LB group was pre-dominantly enriched with specific lineages, including Patescibacteria, Firmicutes B, Desulfobacterota_I, Elusimicrobiota, *Faecousia*, and *Phocaeicola A* (*P* < 0.05). Conversely, microbial taxa significantly overrepresented in the HB group primarily comprised *Faecalimonas, Clostridium_P, Ruminiclostridium_E* (*P* < 0.05).

### 3.5 Serum metabolomics analysis

**Figure 5:**
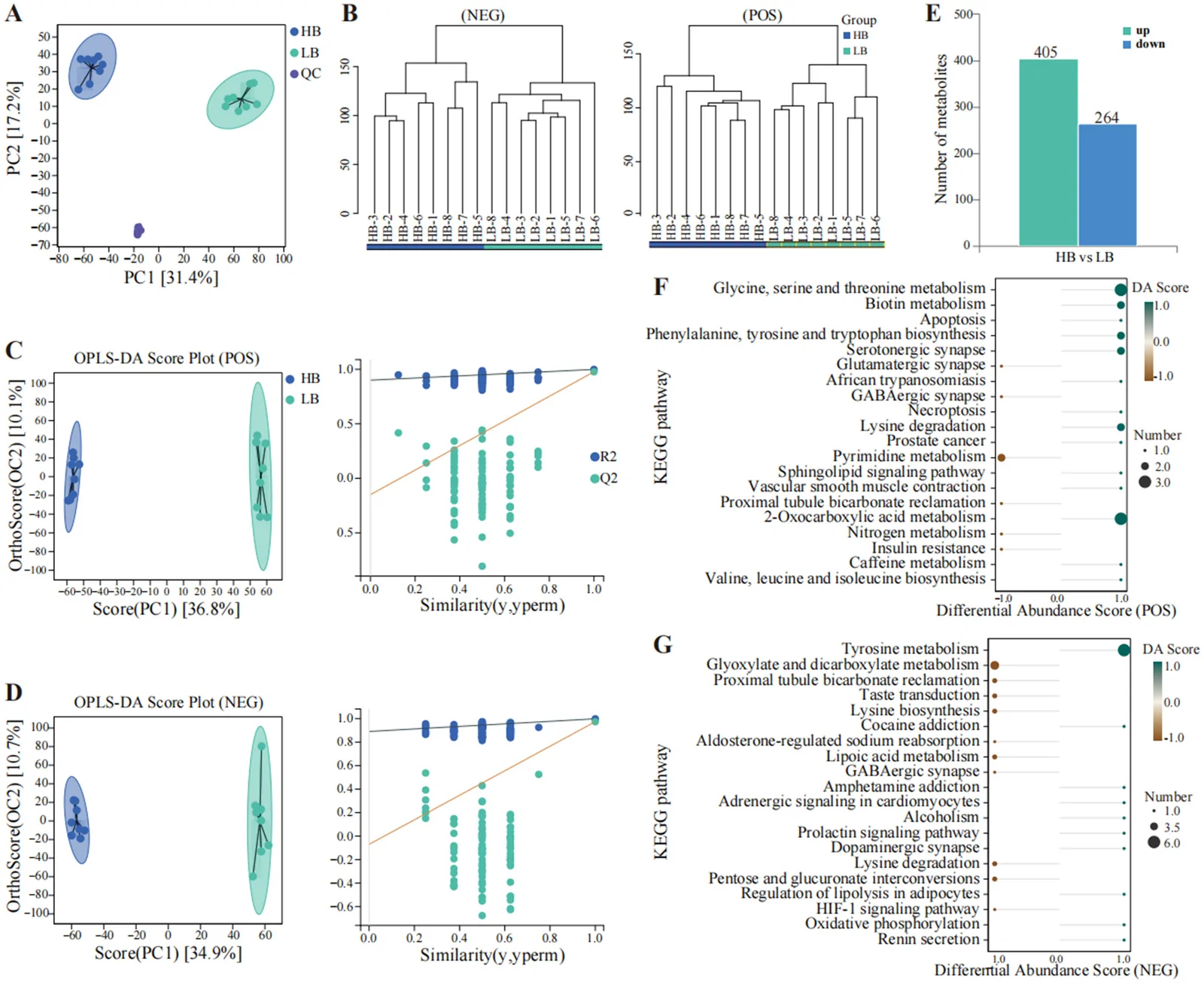
Comparison of serum metabolome of Boer goat at different altitudes. **(A)** Principal component analysis (PCA) score plot; **(B)** Hierarchical clustering analysis; **(C)** OPLS-DA score plot and permutation test (positive ion mode); **(D)** OPLS-DA score plot and permutation test (negative ion mode); **(E)** Number of differential metabolites; **(F)** DA score plot of KEGG pathway enrichment analysis (positive ion mode); **(G)** DA score plot of KEGG pathway enrichment analysis (negative ion mode).

Analysis of serum metabolites showed a clear separation between the HB and LB groups in the PCA, with samples clustering distinctly by altitude of origin, while the QC samples clustered tightly together, confirming data reliability and reproducibility (Figure 5A). Furthermore, hierarchical cluster analysis based on Euclidean distance demonstrated that within both the positive and negative ion modes, the HB and LB samples segregated into distinct clades, indicating high intra-group similarity in metabolic profiles (Figure 5B). To further evaluate the metabolic differences between the HB and LB groups, OPLS-DA analysis was employed. The resulting score plots exhibited clear group separation, demonstrating the model's robust discriminative capability, indicating substantial metabolomic divergence induced by high-altitude adaptation. Model permutation test was statistically confirmed with an R^2^Y value of 0.993 and a *Q*^2^ value of 0.504 in the positive mode, alongside identical metrics (R^2^Y = 0.993, *Q*^2^ = 0.504) in the negative mode (Figures 5C, D). A *Q*^2^ value exceeding 0.5 indicates satisfactory predictive reliability for the OPLS-DA models. From these validated models, a total of 669 reliable differential metabolites were identified in the positive and negative ion modes, respectively (Figure 5E). Subsequently, the identified differential metabolites were subjected to KEGG enrichment analysis (Figures 5F, G). The results indicated that these metabolites were primarily associated with pathways related to energy metabolism, amino acid metabolism, and neurotransmitter signaling. Specifically, significantly enriched pathways included Glycine, serine and threonine metabolism; Biotin metabolism; Serotonergic synapse; GABAergic synapse; Sphingolipid signaling pathway; 2-Oxocarboxylic acid metabolism; Glyoxylate and dicarboxylate metabolism; Dopaminergic synapse; Pentose and glucuronate interconversions; HIF-1 signaling pathway; and Oxidative phosphorylation.

**Figure 6:**
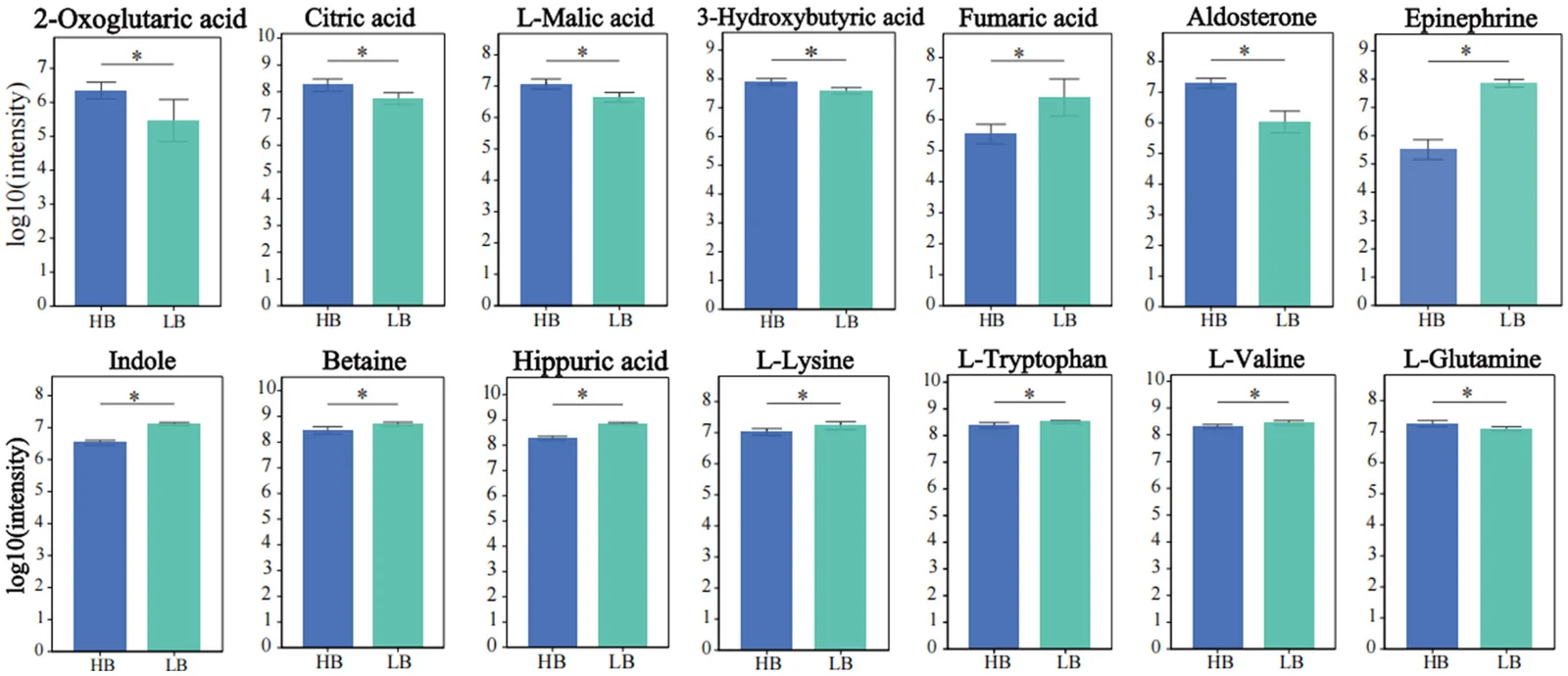
Key differentially serum metabolites of Boer goat at different altitudes. ^*^ indicates a significant difference at P < 0.05.

Focusing on these key KEGG pathways, the specific alterations of key differential metabolites were illustrated in Figure 6. Intermediate metabolites associated with the tricarboxylic acid (TCA) cycle and energy metabolism—specifically 2-oxoglutaric acid, citric acid, L-malic acid, and 3-hydroxybutyric acid—were significantly enriched in the HB group (*P* < 0.05). Conversely, fumaric acid decreased significantly. Regarding neuroendocrine and stress-adaptation markers, aldosterone was markedly upregulated, whereas epinephrine was notably depleted in the HB group (*P* < 0.05). Furthermore, a substantial reduction was observed in the circulating levels of several amino acids and potential microbial co-metabolites, including L-valine, L-tryptophan, L-lysine, betaine, indole, and hippuric acid, in the high-altitude goats. In contrast, circulating L-glutamine levels were significantly elevated (*P* < 0.05).

### 3.6 Correlation analysis

**Figure 7:**
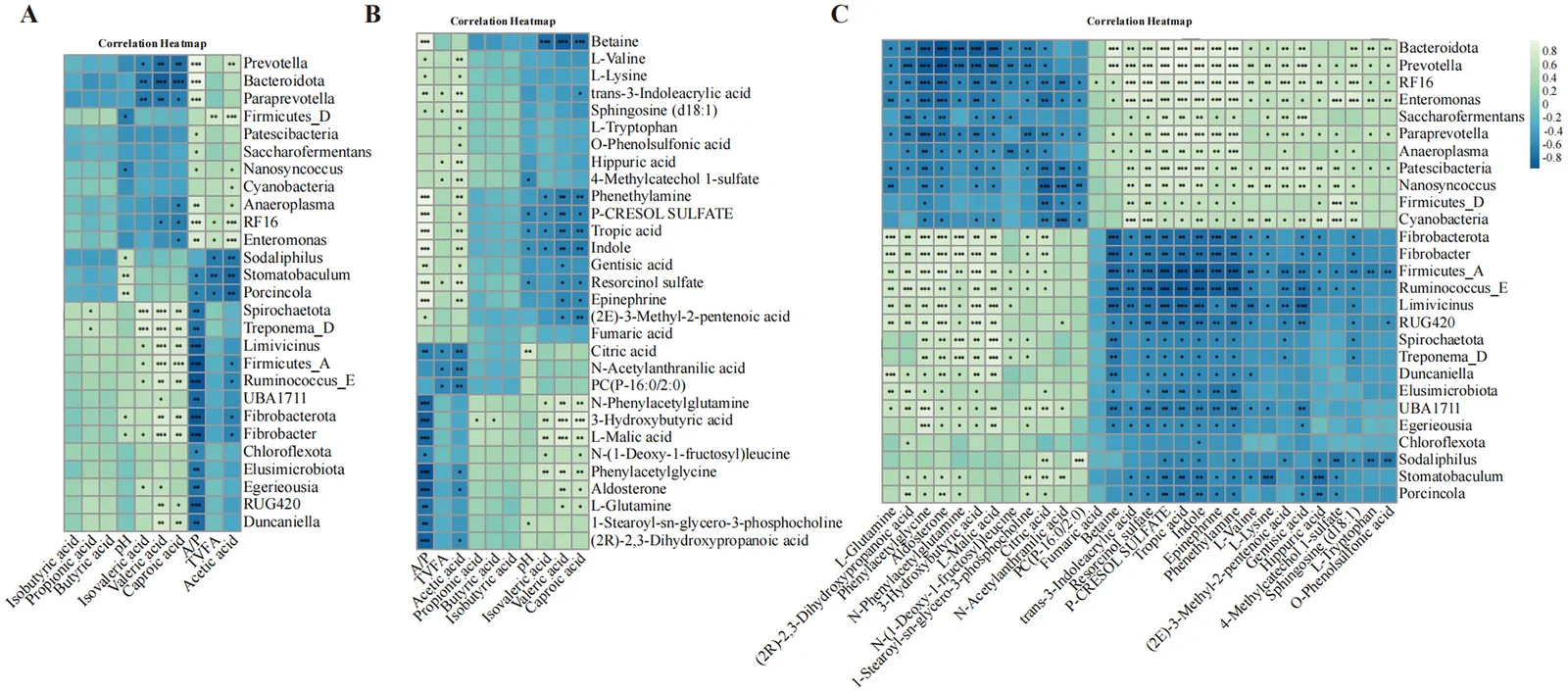
Correlation Analysis. **(A)** Spearman correlation between differentially rumen microorganisms and rumen VFAs; **(B)** Spearman correlation between differentially serum metabolites and rumen VFAs; **(C)** Spearman correlation between differentially rumen microorganisms and differentially serum metabolites.

Correlation analysis between differential rumen microbiota and VFAs (Figure 7A) revealed that Bacteroidota, *Prevotella*, and *Paraprevotella* were significantly and negatively correlated with isovaleric acid, valeric acid, and caproic acid, while exhibiting a strong positive correlation with the A/P ratio (*P* < 0.05). Conversely, Spirochaetota, Firmicutes_A, Fibrobacterota, *Treponema_D, Limivicinu*s, *Ruminococcus_E*, and *Fibrobacter* were inversely correlated with the A/P ratio. Furthermore, Firmicutes_D, *Prevotella, Nanosyncoccus*, Cyanobacteria, Anaeroplasma, and *RF16* displayed robust positive correlations with the A/P ratio. Fibrobacterota, *Ruminococcus_E*, and *Fibrobacter* showed significant negative associations with acetic acid, whereas Spirochaetota and *Treponema_D* were strongly positively correlated with propionic acid (*P* < 0.05).

Regarding the interactions between rumen VFAs and differential serum metabolites (Figure 7B), the circulating levels of betaine, L-valine, L-lysine, trans-3-indoleacrylic acid, and sphingosine (d18:1) were significantly and positively correlated with the A/P ratio (*P* < 0.05). Moreover, phenethylamine, p-cresol sulfate, tropic acid, indole, gentisic acid, resorcinol sulfate, epinephrine, and (2E)-3-methyl-2-pentenoic acid exhibited strong positive correlations with both the A/P ratio and acetic acid, but were significantly and negatively correlated with valeric acid (*P* < 0.05). Additionally, 3-hydroxybutyric acid was positively correlated with propionic acid and butyric acid. In contrast, citric acid, L-malic acid, N-(1-deoxy-1-fructosyl) leucine, phenylacetylglycine, aldosterone, and L-glutamine demonstrated significant negative correlations with the A/P ratio (*P* < 0.05).

The correlation network between differential rumen microbiota and serum metabolites (Figure 7C) further showed the host-microbiome co-metabolic patterns. Specifically, *Fibrobacterota, Fibrobacter*, Firmicutes_A, *Ruminococcus_E, Limivicinus*, and *Treponema_D* were strongly positively correlated with phenylacetylglycine, aldosterone, N-phenylacetylglutamine, 3-hydroxybutyric acid, and L-malic acid. Conversely, these taxa exhibited significant negative correlations with trans-3-indoleacrylic acid, *p*-cresol sulfate, tropic acid, indole, epinephrine, phenethylamine, (2E)-3-methyl-2-pentenoic acid, and sphingosine (d18:1) (*P* < 0.05). Meanwhile, *Bacteroidota, Prevotella*, and *RF16* were significantly and positively associated with L-glutamine, phenylacetylglycine, aldosterone, N-phenylacetylglutamine, 3-hydroxybutyric acid, and L-malic acid. However, these specific taxa were inversely correlated with trans-3-indoleacrylic acid, *p*-cresol sulfate, tropic acid, indole, epinephrine, phenethylamine, (2E)-3-methyl-2-pentenoic acid, sphingosine (d18:1), L-valine, and L-lysine (*P* < 0.05).

## 4 Discussion

Ruminants produce VFAs through fermenting structural carbohydrates (primarily cellulose and hemicellulose) from forage. The VFAs not only serve as primary energy substrates but also stimulate the sympathetic nervous system to modulate energy expenditure (Bergman, 1990; Sha et al., 2022). Previous studies have established that altitude serves as a primary environmental stressor affecting ruminant productive performance; hosts acclimatize by modulating energy intake, optimizing metabolic cascades, and adjusting oxygen and energy expenditure. Within this context, the rumen functions as a critical organ for feed digestion and energy conversion, fostering intricate cross-talk between the resident microbiome, its associated metabolome, and the host to facilitate survival in extreme alpine environments (Cui et al., 2019).

Following the introduction of Boer goats to high-altitude regions, their feeding management transitions from an intensive indoor system to a semi-grazing model. This shift necessitates the ingestion of natural pasture characterized by elevated cellulose content and reduced overall forage quality, thereby challenging nutrient supply. Consequently, the ruminal fermentation pattern is significantly altered to cope with these extreme plateau conditions. Propionate is the pre-dominant VFA in the rumen of high-altitude ruminants, functioning as the fundamental energy source for both the host and the microbiota. In the rumen ecosystem, propionate is pre-dominantly synthesized via the succinate and acrylate pathways, processes driven by a diverse array of specific microbial consortia (Zhao et al., 2020). Importantly, propionate serves as the most critical glucogenic precursor for net glucose synthesis (Allen, 2014). Elevated ruminal propionate concentration is a positive indicator of improved energy utilization and reduced cold stress. A diminished A/P ratio is strongly positively correlated with superior feed energy conversion (Chen et al., 2021). Therefore, it is deduced that high-altitude Boer goats exhibit strong energy utilization efficiency to maintain normal production performance under hypoxic and frigid conditions.

Elevational shifts profoundly modulate the composition and structural dynamics of both ruminal and fecal microbiota. Previous studies have demonstrated that the ruminal microbiome of yaks exhibits distinct altitudinal adaptability, characterized by a significantly higher proportion of fibrolytic bacteria in high-altitude habitats compared to lower elevations (Pan et al., 2024). Consistent with these observations, the current study shows that as altitude increased, the overall diversity of ruminal and fecal microbiota was markedly diminished, accompanied by a significant enrichment of fiber-degrading taxa. Firmicutes is primarily responsible for the degradation of structural carbohydrates and is renowned for its capacity to harvest maximal energy from limited dietary inputs. Bacteroidota pre-dominantly target non-fibrous substrates (Reigstad and Kashyap, 2013). This observation aligns with the findings of Xie et al. (2026) in high-altitude yaks, reflecting a community-level ecological shift toward highly efficient fermentation strategies under conditions of nutrient scarcity. Interestingly, previous studies revealed that the gastrointestinal microbiota of high-altitude mammals exhibits a significantly higher F/B ratio compared to their low-altitude counterparts (Liu et al., 2021). F/B ratio has been robustly proven to facilitate energy harvesting, maintain metabolic homeostasis, and is strongly positively correlated with enhanced fat accumulation and average daily gain (Chen et al., 2015; Min et al., 2019). The present study show that the F/B ratio was markedly elevated in Boer goats following their introduction to the Tibetan Plateau. Consequently, the elevation of the F/B ratio signifies an augmented adaptive capacity of high-altitude Boer goats against severe winter cold stress.

Furthermore, Fibrobacterota serves as a keystone fibrolytic phylum in the ruminant rumen, empowering the host to effectively depolymerize alpine forage and generate vital energy metabolites (Ransom-Jones et al., 2012; Sha et al., 2023). *Ruminococcus* plays a pivotal role in the biodegradation of resistant starch and other dietary fibers (Ze et al., 2012). Ruminococcaceae and Lachnospiraceae function as major short-chain fatty acid-producing microbial families. These taxa are intimately associated with cold stress adaptation, energy retention, and overall feed conversion efficiency in ruminants (Guo et al., 2021; Fan et al., 2021). In the present study, the relative abundances of Ruminococcaceae, Fibrobacterota, and Fibrobacter were strongly and negatively correlated with the A/P ratio. Consequently, we postulate that the over represented Firmicutes, Fibrobacter and Ruminococcaceae in the rumen of high-altitude Boer goats actively mediate the degradation of cellulosic substrates. This microbial shift likely empowers the host to efficiently extract dietary energy in harsh environments, thereby sustaining overall energy homeostasis and core body temperature.

Interestingly, although the rumen microbiota and its associated VFA profile underwent significant remodeling following high-altitude exposure, it is noteworthy that there were no significant differences in fecal VFA concentrations among the different altitude groups. This phenomenon is fully consistent with the physiological mechanisms of ruminant digestion, namely that the rumen is the primary site for the fermentation of structural carbohydrates and the absorption of VFAs. The absence of significant changes in fecal VFA concentrations may suggest that energy acquisition in Boer goats is largely dominated by the upper digestive tract. Furthermore, VFAs produced by hindgut fermentation are likely absorbed by the host with maximum efficiency to meet the substantial energy demands imposed by cold stress. Therefore, the microbial communities in the rumen, rather than those in the hindgut, are the primary determinants of Boer goats' systemic metabolic adaptation to the high-altitude environment.

Under grazing conditions, survival in the relatively harsh alpine environment necessitates substantial endogenous energy expenditure for high-altitude Boer goats. Severe hypoxic and cold stress inevitably lead to the depletion of serum glucose and hepatic glycogen, creating a substantial energy deficit for the host. As serum metabolomic profiles show, high-altitude adaptation involves clear changes in energy and amino acid metabolism, coupled with water-salt balance regulation and neurobehavioral adaptations. The significant upregulation of specific metabolites—such as L-malic acid, 2-oxoglutaric acid, 3-hydroxybutyric acid, and citric acid—in the high-altitude group supports this metabolic shift. Notably, 2-oxoglutaric acid is involved in the HIF-1 signaling pathway—a central regulatory hub for cellular hypoxia adaptation. As a key co-substrate for prolyl hydroxylases (PHDs), 2-oxoglutaric acid determines the stability and degradation process of hypoxia-inducible factor 1-alpha (HIF-1α) (Xiong et al., 2018). These metabolites are intrinsically involved in crucial energy-related pathways, including the TCA cycle, glucagon signaling pathway, glyoxylate and dicarboxylate metabolism, thereby facilitating physiological acclimatization to the extreme environment. Their marked enrichment indicates an accelerated turnover of energy metabolic pathways to compensate for the hypoxia-induced energy shortfall (Chicco et al., 2018; Liu et al., 2023). Intriguingly, the present study show that 3-hydroxybutyric acid is strongly positively correlated with propionic acid. Following the introduction of goats to high altitudes, the ruminal propionic acid concentration is significantly enriched. Upon entering the liver, propionic acid is converted into succinyl-CoA, which subsequently enters the TCA cycle (Longo et al., 2017). The upregulation of serum TCA cycle intermediates and ruminal propionic acid suggests that ruminal fermentation efficiency is well maintained. Furthermore, the strong positive correlations between these TCA intermediates and fibrolytic taxa (e.g., Fibrobacterota, *Fibrobacter*, Firmicutes_A, and *Ruminococcus_E*) highlight the importance of host-microbiome co-metabolism in energy adaptation, allowing Boer goats to efficiently convert feed into energy under high-altitude conditions.

To fulfill the high energy demands imposed by the plateau environment, amino acid catabolism plays a crucial role in fueling the TCA cycle. This is supported by the enrichment of the alanine, aspartate, and glutamate metabolic pathways, especially the upregulation of L-glutamine. Glutamine is converted to glutamate by glutaminase and oxidized to α-ketoglutarate in its entry into the TCA cycle (Ta and Seyfried, 2015). Polet and Feron (2013) discovered that under hypoxic conditions, glutamine can provide nutrients for the TCA cycle in angiogenic endothelial cells. Additionally, maintaining the homeostasis of extracellular fluid through the autoregulation of water-salt metabolism is crucial for high-altitude adaptation (Toshboltaeva and Murtazina, 2011). In the steroid hormone biosynthesis pathway, aldosterone promotes sodium retention in the kidneys and maintain the intravascular fluid volume, thereby ensuring water-salt balance and maintaining the relative stability of the internal environment within the body (Sun et al., 2013).

Furthermore, high-altitude causes significant neurobehavioral adaptations mediated by tyrosine and phenylalanine metabolisme, GABAergic synapses. Tyrosine metabolism has been implicated in myocardial ischemia–reperfusion, pulmonary hypertension and adaptability to high-altitude stress (Zhao et al., 2025). The significant modulation of L-DOPA and epinephrine indicates the presence of a classical sympathoadrenal stress response, which is essential for optimizing cardiac output and metabolic reserves during chronic hypoxia (Maini et al., 2019; Richalet, 2016). Previous studies show that plasma epinephrine concentrations in high-altitude mice remain substantially lower than those in low-altitude cohorts under both normoxic and hypoxic conditions (Pranckevicius et al., 2025). Thus, the lower epinephrine levels observed in high-altitude Boer goats likely reflect a neuroendocrine adaptation to the plateau environment. Additionally, L-tryptophan and its microbial co-metabolite, indole, exhibited a remarkable downward trend at high altitudes. A variety of microorganisms in the gastrointestinal tract jointly participate in the tryptophan-indole metabolic pathway. These findings suggest that microbiota-driven alterations in tryptophan and indole likely help regulate host high-altitude acclimatization by modulating neurotransmitter release, immune responses and systemic metabolism (Su et al., 2022).

Integrating the multi-omics data reveals a clear rumen-host metabolic cross-talk that is essential for the high-altitude adaptation of Boer goats. Driven by the high-altitudes environment and high-fiber forage, the ruminal microbiome underwent a reshaping characterized by a significant enrichment of fiber-degrading bacteria (such as Fibrobacterota and *Ruminococcus_E*). This shift directs the ruminal fermentation toward a propionate-dominant profile, resulting in a diminished A/P ratio. Upon absorption, the increased propionate serves as a major glucogenic precursor. Concurrently, serum metabolomics revealed significant accumulation of TCA cycle intermediates (e.g., L-malic acid, citric acid) and 3-hydroxybutyric acid. Furthermore, strong correlations between specific taxa (e.g., *Prevotella*) and serum neuro-immunomodulatory metabolites (e.g., indole derivatives, L-tryptophan) highlight the extensive scope of host-microbiome co-metabolism. However, since our current data only indicates correlations, further functional experiments are necessary to verify the causal relationships between specific microbes and host metabolic changes. In summary, these findings suggest that co-metabolism between the host and the microbial community likely contributes to energy adaptation, potentially serving as a biological mechanism that helps Boer goats withstand severe plateau stress.

However, the metabolic and microbial shifts observed in the high-altitude group cannot be exclusively attributed to hypoxic stress. The semi-housing management and the intake of natural, high-fiber pasture also exert effects on ruminal fermentation and host metabolic profiles. Therefore, the elevated TCA intermediates reflect a synergistic adaptation. While the HIF-1 signaling pathway provides clear evidence of a physiological response to low oxygen, the different diets simultaneously shape the ruminal microbiota and alter energy precursors. Our current data only indicate correlations rather than causations. To better understand these complex adaptive mechanisms, future studies should strictly control environmental variables throug standardized feeding trials to separate the independent effects of diet and hypoxia. Meanwhile, given the lack of a time-course sampling gradient in the present study, it is difficult to distinguish whether the multi-omics alterations stem from long-term high-altitude acclimatization. Therefore, subsequent research should incorporate multiple sampling time points to observe the dynamic patterns of high-altitude adaptation. Furthermore, functional experiments including *in vitro* fermentation and microbiota transplantation are highly necessary to verify the causal relationships between specific microbes and host metabolic changes.

## 5 Conclusions

This study shows that the introduction of Boer goats to high-altitude environments is accompanied by coordinated changes in the rumen microbiome and host metabolism. The ruminal microbiota shifts toward a propionate-dominant fermentation profile enriched with fiber-degrading taxa (such as Fibrobacterota and *Ruminococcus_E*). Concurrently, systemic metabolic alterations—particularly in energy and amino acid metabolism, hydromineral balance, and neurotransmitter regulation—likely help the host meet the energy demands of the plateau. In summary, the metabolic synergy between the rumen microbiome and the host likely contributes to maintaining energy homeostasis in high-altitude environments. These findings provide a useful reference for developing targeted nutritional strategies and improving livestock management in highland ecosystems.

## Data Availability

The data analyzed in this study is available in the Genome Sequence Archive (GSA) (https://ngdc.cncb.ac.cn/gsa) under accession number CRA048365.
