# Supplementary material for: Integrative analysis of rumen microbiome and host metabolism reveals adaptive strategies of Boer goats to high-altitude environments
Source: Front Microbiol. 2026 Aug 25;17:1897971. doi: 10.3389/fmicb.2026.1897971 (PMC13552352; doi:10.3389/fmicb.2026.1897971)
Supplement: Supplementary file 1 [file Data_Sheet_1.docx]

**Supplementary material**

**Table S1**

*Categories, their definition, frequencies, and examples of words/expressions*

| **Categories** | **Definition** | Frequency | Examples |
| --- | --- | --- | --- |
| **Police and security forces** | Related to police and other security forces responsible for maintaining public safety, enforcing laws, and protecting citizens from direct threats. | 3140 | police force, municipal police, good police, federal police, strengthen the police, lack of police, to the police, police training, police reinforcement, police protection, police control, police, police operation |
| **Law and order** | The framework of laws and institutions that ensure justice, regulate behaviour, and preserve social stability. | 2121 | laws, rules, law, court, laws, law and order, lawyer, the law, lawful, new laws, laws and regulations, law that protects, |
| **Safety and security** | Emotional need | 2002 | security, protection, safety, safe, securing, ensure security, safety, secure, protected, I feel protected in the country, safety/security, protect, protect, protective, protection systems, spaces, protection policy |
| **Criticism** | The expression of negative evaluation, through criticism. | 1667 | fail, failure, incompetence, liar, inefficiency, negligence, unfair, useless, poor, chaos |
| **Military** | Related to the armed forces of a state responsible for national defence. | 1617 | army, soldiers, armed forces, military service, navy, tanks, artillery, fighter jets, military training, armament |
| **Healthcare and well-being** | Services and initiatives that promote physical and mental health, prevent illness, and enhance quality of life. | 1120 | healthcare, doctors, nurses, hospitals, medical care, public health, NHS, healthcare access, medical services, health system |
| **Politics** | The activities, political figures, and institutions - such as parties - related to politics | 1036 | Government, Parliament, elections, politicians, political parties, Prime Minister, Ministry, voting, campaign, political decisions |
| **Crime** | Acts that violate the law and are punishable by legal authorities. | 848 | crime, violence, theft, fraud, corruption, cybercrime, drug trafficking, assault, murder, organized crime, child abuse, femicide |
| **Welfare** | Government programmes and social policies are aimed at supporting individuals’ basic needs and improving living standards. | 725 | welfare, social welfare, welfare benefits, social security, pensions, social services, unemployment subsidies, subsidies, income support, welfare state |
| **Economy and finances** | The system of production, distribution, and consumption of goods and services, including financial management and policy. | 669 | economy, economic growth, financial security, inflation, banks, investment, budget, taxes, income, market economy |
| **Insecurity** | Everything related to general mentions of insecurity. Negative axis of the emotional need "safety and security". | 657 | insecurity, unsafe, no security, lack of protection, not safe enough, unprotected, weak protection, insufficient protection, vulnerable, no safety |
| **Immigration** | Related to the movement of people into a country to live or work. | 479 | immigration, migrants, immigrants, illegal immigration, migration policy, Immigration control, visas, foreigners, illegal migration, asylum |
| **Defence** | The protection of a nation’s territory, people, and interests through military and strategic means. | 466 | defence, national defence, defence policy, defence spending, civil defence, defence strategy, defence system, Ministry of Defence |
| **Surveillance and monitoring** | The systematic observation and recording of activities, data, and related equipment. | 437 | surveillance, monitoring, CCTV, cameras, video surveillance, facial recognition, tracking, screening, supervision, oversight |
| **Border and territorial security** | The protection and management of a nation’s boundaries to prevent illegal entry, trafficking, and territorial threats. | 382 | border control, border security, border patrol, border protection, border checks, border guards, border surveillance, border policy, coast protection, borders |
| **Environment and climate** | The natural surroundings and atmospheric conditions that affect life on Earth, the threats they pose, and the efforts to preserve and protect them. | 360 | environment, climate change, environmental protection, pollution, renewable resources, nature protection, biodiversity, emission reduction, water protection, climate policy |
| **Positive** | Expressions of general positive evaluation. | 357 | good, excellent, effective, reliable, fair, successful, strong, efficient, positive, quality |
| **Equality, diversity, and inclusion** | EDI - Related to ensuring fair treatment for all individuals regardless of background. | 353 | equality, equal rights, diversity, inclusion, gender equality, equal opportunities, equity, integration, social inclusion |
| **Employment** | Employment-related topics | 341 | employment, jobs, work, job security, workplace safety, workers' rights, labour market, employment protection, unions, employee rights |
| **Emergency and rescue** | Actions and services aimed at responding to urgent situations, saving lives, and providing immediate assistance in crises or disasters. | 338 | emergency services, ambulance, fire brigade, rescue, disaster, crisis management, civil protection, emergency number, first aid, emergency response |
| **Education** | Related to teaching, learning, and acquiring knowledge, skills, and values through schools, institutions, or formal, non-formal, and informal settings. | 331 | education, schools, teachers, learning, training, higher education, public education, education policy, literacy, school system |
| **Child and childcare** | Services, policies, and responsibilities focused on the development, protection, and well-being of children. | 328 | children, child protection, childcare, child welfare, child support, children's rights, youth services, safeguarding children, early childhood |
| **Help and support** | Assistance asked for or provided. | 320 | help, support, assistance, aid, social support, public support, mutual aid, legal support, humanitarian aid, community help |
| **Foreign policy** | Related to the nation’s strategies, multilateralism, and actions in its relations with other countries, including diplomacy, trade, and security. | 309 | diplomacy, international cooperation, NATO, European Union, United Nations, foreign policy, alliances, treaties, international relationships, sanctions against Russia |
| **Guards and safeguarding** | Related to the mentions of guarding and safeguarding. | 304 | guards, security guards, safeguarding, guard, guardianship, bodyguard, Home Guard, coast guard, guarding, safeguard |
| **Punishment** | Related to penalties or sanctions imposed in response to wrongdoing or violation of laws or rules. They can be light, moderate, or harsh. | 292 | punishment, sentencing, penalties, arrest, detention, conviction, sanctions, prison, fines, death penalty |
| **Insurance** | Related to a guarantee of protection against loss, damage, illness, or liability in exchange for regular payments. | 274 | insurance, health insurance, life insurance, social insurance, unemployment insurance, insurance policy, insurance premiums, property insurance, motor insurance, medical insurance |
| **Human rights** | The fundamental freedoms and entitlements that belong to every person, regardless of nationality, gender, or background. | 270 | human rights, fundamental rights, civil rights, rights and freedoms, equality and human rights, right to legal aid, citizen rights, right to health, protection of human rights |
| **Control and order** | The maintenance of discipline, structure, and authority to prevent disorder or instability within a group or society. | 265 | order, public order, control, regulation, supervision, access control, keeping order, authority, restraint, control of people |
| **Housing and rent** | The provision, occupation, use, and environment of accommodation. | 262 | housing, affordable housing, rent, tenant protection, social housing, housing assistance, public housing, housing rights, accommodation, housing policy |
| **Imprisonment and parole** | The confinement of individuals as a punishment and the conditions under which early release may be granted. | 258 | imprisonment, prison, jail, prison sentences, detention, parole, correctional facilities, custody, secure prisons, prison reform |
| **Freedom** | The right or ability to act, speak, or think without undue restraint or interference. | 251 | freedom, liberty, freedom of speech, freedom of expression, freedom of religion, freedom of movement, freedom of assembly, personal freedom, free choice, press freedom |
| **Weapon related** | Matters involving the possession, use, regulation, or impact of weapons and armaments. | 248 | weapons, guns, knives, ammunition, weapon license, firearms, pepper spray, weapon ban, gun permits, armed civilians |
| **Data** | Related to data and data protection. | 206 | data protection, personal data, privacy, GDPR, data security, data breach, cookies, sensitive data, digital data, data privacy |
| **War** | Armed conflict between states and/or groups. | 179 | war, civil war, war in Ukraine, war prevention, wartime preparedness, protection against war, anti-war, war policy, neutrality in war, prevention of war |
| **Policies** | Courses of action adopted by governments, organisations, or individuals to guide decisions and achieve objectives. One of the streams related to policy windows. | 172 | policies, government policies, protective policies, security policies, social policies, regulatory policy, policy, safety policies, fiscal policy, legal policy |
| **Probability and doubt** | The assessment of likelihood and uncertainty when evaluating outcomes, events, or claims. | 172 | certainty, uncertainty, uncertain, unsure, not sure, possible, probably, feasibility, predictability, risk |
| **Peace** | A state of harmony and the absence of conflict, violence, or war within and between societies. | 171 | peace, peace agreements, peacekeeping, ceasefire, peace negotiations, social peace, world peace, peace policy, peaceful, peace talks |
| **Geographical spaces** | Defined areas of land or territory characterised by specific physical, cultural, or political features - such as cities, countries and other places. | 160 | city, country, region, territory, cities, towns, public places, countries, urban centres, suburbs |
| **Diseases and medical conditions** | Illnesses or disorders that affect the normal functioning of the body. | 155 | disease, illness, diseases, sickness, cancer, chronic diseases, infections, disease screening, occupational diseases |
| **Discrimination** | The unfair or unequal treatment of individuals or groups based on characteristics such as race, gender, age, or religion. | 155 | discrimination, less discrimination |
| **Income and purchasing power** | The financial resources available to individuals and their ability to buy goods and services. | 151 | income, wages, salaries, purchasing power, cost of living, prices, savings, minimum wage, wealth, expenses |
| **Democracy** | A system of government in which power is vested in the people, who exercise it directly or through elected representatives. | 147 | democracy, democratic, democratic elections, democratic decisions, democratic rules, lack of democracy, anti-democratic, separation of powers, democracy laws, democratic system |
| **Asylum and refugee** | Related to individuals fleeing persecution, and the status given to those recognised as refugees under international law. | 145 | asylum, refugees, asylum seekers, refugee protection, asylum policy, residence permit, refugee rights, refugee support, political asylum, refugee |
| **Intelligence and counterintelligence** | Related to institutions responsible for the collection and analysis of information for national security purposes and efforts to prevent espionage or infiltration. | 138 | intelligence services, espionage, counterespionage, secret services, intelligence, CIA, MI6, FBI, Shin Bet, Mossad |
| **Importance** | The quality of being significant, valuable, or worthy of attention. | 137 | important, necessary, relevant, required, essential, priority, fundamental, valuable, urgent, vital |
| **Deportation and repatriation** | Related to the process of removing individuals from a country or returning them to their place of origin. | 136 | deportation, deportations, repatriation, expulsion, deport criminals, deport foreign criminals, deport offenders, expel dangerous people to their countries of origin, deport refugees, deportation order |
| **Food and hunger** | The availability, access, and consumption of nourishment, and the challenges related to scarcity or malnutrition. | 135 | food, hunger, food prices, food supply, food security, food safety, food bank, providing food, affordable food prices, access to food |
| **Precautions and prevention** | Actions taken in advance to reduce risks, avoid harm, or prevent negative outcomes. | 132 | prevention, precautions, prevent, preventive measures, risk prevention, preventive work, prevention measures, prevent abuse, mitigation |
| **Family and friends** | Close personal relationships. | 128 | family, families, friends, friendship, parents, mother, father, family support, family protection, loved ones |
| **Regulation and guidelines** | Mentions to regulations, procedures, guidelines, and instructions. | 127 | regulations, regulation, guidelines, guideline, procedures, instructions, legal clause, regulate, safety guidelines, public regulations |
| **Fear** | Emotion | 123 | fear, scared, afraid, scary, frightening, concern, worry, panic, fear propaganda, in fear |
| **State** | The political and administrative organisation of a nation, exercising authority over its territory and population. | 123 | state, the state, state, state security, state protection, state bodies, state organizations, state function, state ownership, state integrity |
| **Air security and defence** | Related to operations designed to protect airspace and prevent airborne threats or attacks. | 122 | air defence, air force, drones, missiles, airspace, anti -missile systems, Iron Dome, David’s sling, Arrow 3, aerial protection |
| **Alert and alarm** | Warnings or notifications indicating danger, requiring attention or immediate action. | 115 | alert, alarm, alerts, warning, warning system, sirens, panic buttons, early warning system, emergency alerts, warning signs |
| **Fire and firefighters** | The occurrence of uncontrolled flames and the personnel and services dedicated to extinguishing them and ensuring safety. | 113 | fire, firefighters, fire brigade, fire protection, fire prevention, fire service, fire extinguisher, fire department, firefighter, firefighting |
| **Plans, action, and execution** | The processes of preparing, implementing, and completing strategies, interventions, or operations. | 112 | action, actions, action plan, plans, plan, implementation, execution, intervention, response, strategy |
| **Caring** | Mentions to care and taking care. | 110 | care, caring, take care, taking care of people, care for citizens, care for others, special care, duty of care, care agency, care centre |
| **Terrorism** | The use or threat of violence to instil fear and achieve political, ideological, military, or religious goals. | 110 | terrorism, terrorists, terrorist, counterterrorism, anti-terrorism, preventing terrorism, protection against terrorism, anti-terror units, terrorist attacks, terror prevention |
| **Cohesion and solidarity** | Emotional need | 101 | solidarity, unity, cohesion, fraternity, togetherness, union, shared responsibility, fellowship, common task, join |
| **Confidence** | The belief in one’s abilities, or the trust placed in people, systems, or outcomes. | 101 | confidence, confident, personal confidence, political confidence, civilian confidence, confidence in the streets, confidence in the community, feel confident, self-confidence, inspiring confidence |
| **Road and cars safety** | Related to road and car safety. | 101 | road safety, road, roads, traffic, traffic rules, speed limit, driving, seat belts, road accidents, traffic control |
| **Commitment and responsibility** | The obligation or dedication to perform duties, uphold values, or fulfil promises. | 99 | responsibility, commitment, duty, duties, take responsibility, personal responsibility, social responsibility, public responsibility, obligation, engagement |
| **Shelter** | A place providing protection or refuge from danger, weather, or hardship. | 99 | shelter, shelters, bomb shelter, nuclear shelter, safe rooms, emergency shelters, women's shelter, protection room, shelter for battered women, anti-atomic shelters |
| **People** | Human beings collectively are considered members of a community, nation, or group. | 95 | people, population, citizens, person, individuals, inhabitants, the people, persons, residents, public |
| **Infrastructures** | The physical and organisational structures needed for the operation of a society, such as transport, energy, and communication systems. | 94 | infrastructure, critical infrastructure, infrastructure in place, public lighting, street lighting, buildings, streets, energy generators, bridge, dams |
| **Privacy** | The right or condition of being free from unwanted observation, intrusion, or disclosure of personal information. | 94 | privacy, privacy policy, privacy protection, personal data, confidentiality, private life, right to privacy, data privacy, privacy concerns, medical confidentiality |
| **Communication** | The exchange of information, ideas, or feelings between individuals or groups. | 93 | communication, communications, communicative, dialogue, expression, voice, words, talk, discussion, conversation |
| **Social** | Relating to society. | 92 | social, socialness, social society, social stability, social togetherness, social divides, social network, social net, social sponsorship, social groups |
| **Digital and technology** | Systems, devices, and innovations based on digital processes, computing, and information technology. | 87 | digital, digitization, technology, technologies, internet, social media, AI (Artificial Intelligence), VPN (Virtual Private Network), GPS (Global Positioning System), online |
| **Elder and elderly care** | Support and services are provided to older adults to maintain health, dignity, and quality of life. | 86 | elderly, elderly care, elderly support, elderly protection, seniors, senior, old age, ageing population, help seniors, elderly care homes |
| **Investigation, search, and detection** | The process of examining, exploring, or discovering information to uncover facts or solve problems. | 86 | investigation, inspection, search, detection, track, tracking, checks, checkpoints, security check, passport checks |
| **Harm, danger, and risk** | The potential for injury, damage, or loss resulting from exposure to hazards or threats. | 85 | danger, dangerous, risk, risky, threat, threats, harm, harmful, protection from harm, external threats |
| **Force and firmness** | The application of strength, or determination to achieve control or compliance. | 84 | force, strength, strengthen, strong, stronger, firmness, firm, strict, pressure, dominance |
| **Barriers** | Obstacles that prevent progress or access. | 82 | barrier, barriers, fence, fences, wall, checkpoints, boundaries, fortifications, separation fence, ring-fence |
| **Religion** | A system of beliefs and practices centred on faith, spirituality, and moral values. | 81 | religion, God, Christianity, Islam, church, mosques, religious organisations, prayers, Torah study, yeshiva |
| **Mental and psychological** | Relating to the mind and psychology in general. | 80 | mental health, mental health care, psychiatry, therapy, psychological support, mental illness, psychological help, trauma, emotional, behaviour |
| **Trust** | Emotion (Plutchik) - Reliance on the integrity, ability, or character of a person or institution. | 80 | trust, trustworthy, trusted, trust in the state, trust in government action, reliance, trust society, trust the state, trusted environment, self-trust |
| **Calmness** | Emotion | 79 | calm, calmness, calming, keep calm, composure, relaxation, relaxed, serenity, stress-free, not worried |
| **Confusion and vagueness** | A lack of clarity or understanding. | 75 | confusion, confusing, confused, unclear, vague, doubt, doubtful, doubts, misunderstood, nonsense |
| **Unemployment** | The state of being without paid work while actively seeking employment. | 75 | unemployment, unemployment benefits, unemployment support, unemployment subsidy, unemployed, unemployment allowance, unemployment fund, unemployment insurance, support for the unemployed, reduce unemployment |
| **Citizenship and civilians** | Individuals within a nation, and people not engaged in military service. | 73 | citizens, citizen, citizenship, civilians, civilian, citizen protection, citizens' interests, protect citizens, citizen security, citizens' safety |
| **COVID-19** | The infectious disease caused by the SARS-CoV-2 virus led to a global pandemic beginning in 2019. | 73 | covid, covid-19 (pandemic disease), pandemic, vaccination, masks, quarantine, lockdown, epidemic, testing, disinfectant |
| **Cognitive** | Related directly to cognition. | 72 | thinking, reason, intelligence, smart, knowledge, wisdom, common sense, reflection, focus, conscious |
| **Patrols** | The act of monitoring or inspecting an area to ensure security and safety. | 72 | patrol, patrols, patrolling, police patrol, night patrol, neighbourhood patrol, road patrol, on patrol, patrol service, patrols in the city |
| **Equipment** | Tools, devices, or machinery necessary for specific tasks or activities. | 70 | equipment, protective equipment, PPE (Personal Protective Equipment), gas mask, armour, handcuffs, riot gear, helmet, shield, bulletproof vest |
| **Women** | Women-related. | 68 | women, women's rights, women's protection, protection of women, support for women, safe place for women, women’s aid, battered women, violence towards women, women's security |
| **Property** | Anything owned by an individual or entity. | 67 | property, property protection, property rights, private property, public ownership, ownership, confiscation of property, intellectual properties, copyright, goods/property |
| **Poverty and homelessness** | The condition of lacking financial resources and stable housing is necessary for a basic standard of living. | 63 | poverty, homelessness, homeless, anti-poverty, fight against poverty, help for the poorest, housing and homelessness aid, prevention of homelessness, poor people, urban poverty |
| **Respect** | Consideration and regard for the feelings, rights, and values of others. | 63 | respect, respected, respectful, mutual respect, respect for others, treat people with respect, consideration, considerate, openness and respect, respect for everyone |
| **Upgrade and improve** | To enhance the quality, efficiency, or effectiveness of something through change or innovation. | 62 | upgrading, to improve, can be improved, improve, improving, enhance, enhancement, upgrade, update, improvement/refinement |
| **Life** | The existence and experience of living beings encompass biological, social, and emotional aspects. | 61 | life, protection of life, right to life, dignified life, life assurance, life support, life protection, everyday life, lifestyle, lives |
| **Limit** | A boundary or restriction. | 61 | limit, limited, limitations, limits, limiting, boundary, confidence in boundaries, set the boundaries, limitation, the limit |
| **Power** | The ability or capacity to influence, control, or direct people, events, or outcomes. | 61 | power, handling power, power to the people, the power elite, can, empowerment, give more power, powerless, authority/power, abuse of power |
| **Information** | Knowledge communicated or received about facts, events, or circumstances. | 59 | information, personal information, public information, citizens’ information, necessary information, information policy, information security, credible information, information to the population, inquiry |
| **Negligence and abandonment** | The failure to provide or receive necessary care or attention, resulting in neglect or desertion. | 58 | neglect, neglected, neglecting minorities, abandonment, feeling of abandonment, abandoned, abandonment of hostages, negligence, forgotten, do not abandon citizens |
| **Right** | Focused on the lemma, right with vague words/expressions. | 58 | right, all right, guarantee of the right, our right, only right, right -right, only right, right, is it right, grandmas against right |
| **Institution** | An established organisation or system with a specific purpose. | 56 | institution, institutions, the institution, protection of institutions, organs (or bodies, e.g., institutions), bodies, social bodies |
| **Aggression, provocation, combat, and vengeance** | Hostile or violent behaviour driven by conflict, retaliation, or provocation. | 55 | attacks, attack, attacking, preventing attacks, aggression, offensive initiatives, revenge, vengeance, combat, provocation |
| **Complex and difficult** | Complicated, challenging, or hard to resolve or understand. | 52 | difficult, complex, difficulty, complicated, complexity, hard, challenging, not simple, demanding, tricky |
| **Consumers and sellers** | Individuals or entities involved in the buying and selling of goods and services within the market. | 52 | consumer rights, consumer protection, consumers, consumer, consumer ombudsman, consumer protection law, consumer protection authority, buyer protection, rights of sellers, customer |
| **Document** | A written, printed, or digital record that provides information or serves as evidence of actions or decisions. | 52 | documents, document, documentation, passport, passports, ID, visas, visa policy, passport control, papers |
| **Authorities** | Organisations or officials with the power to enforce laws, make decisions, or administer public affairs. | 50 | authorities, authority, authority presence, local authorities, the authorities, reliable authorities, security authorities, executive, supervisory authorities, hierarchy |
| **Vulnerability** | The state of being exposed to harm, risk, or exploitation due to weakness or lack of protection. | 49 | vulnerability, vulnerable, vulnerable persons, vulnerable adult, vulnerable adults, safety for the vulnerable, protection of vulnerable adults, support vulnerable groups, protection of weak, vulnerable people |
| **Comparison and change** | The act of comparing similarities and differences, and the process of transformation. | 48 | changes, change, needs change, reduction, different, same, difference, decreasing, decrease, normal |
| **Question** | All the entries with questions. | 48 | What will happen here? Why? Who is that?, which protective measures?, who protects us?, protection from what?, is there anyone?, can we manage?, where is the information, what is it? |
| **Violence - general** | Behaviour involving physical force intended to harm, damage, or intimidate others. | 48 | violence, acts of violence, violence prevention, protection against violence, anti-violence, no violence, non-violence, protection from violence, violent, violent adult |
| **Media** | Channels of communication, such as newspapers, television, and online platforms, that disseminate information to the public. | 46 | media, journalism, news, radio, TV, interview, the freedom of the media, television, Danish Radio, independent media |
| **Community** | A group of people sharing common interests or identity. | 45 | community, communities, support community resilience, the community, community protection, empower communities, good community, community support, community watch, community unity |
| **Disable and disability** | Conditions that limit physical, mental, or sensory functioning, and the social context surrounding them. | 45 | disabled, disabled people, disability, disabilities, disability support, disability rights, disability protection, support for disabled people, disability allowances, people with disabilities |
| **Skills and abilities** | The capacities and competencies enabling individuals to perform tasks effectively. | 44 | ability, openness, patience, creativity, professionalism, assertiveness, loyalty, compassion, competence, pragmatism |
| **Disrespect** | Behaviour showing a lack of regard, courtesy, or consideration for others. | 43 | disrespect, disrespectful, lack of respect, disrespectful in human life, the leadership's disrespect for the ordinary citizen |
| **Stability** | The condition of being steady, consistent, and resistant to sudden change or disruption. | 43 | stability, stable, stabilization, gives stability, maintain stability, maintaining stability, stand stable, regional stability, no stability |
| **Transportation** | Systems and means of moving people or goods from one place to another. | 43 | transport, transportation, electric vehicles, boats, planes, car, bus, trains, railway protection, public transport |
| **Vaccination** | The administration of a vaccine to protect individuals and communities from infectious diseases. | 42 | vaccination, vaccinations, vaccine, vaccines, vaccinate, vaccination protection, vaccine program, mandatory vaccinations, free vaccinations, protective vaccinations |
| **Good intentions and common good** | Actions motivated by positive aims and the desire to benefit society as a whole. | 41 | common good, good intentions, goodwill, kindness, kind, well-intentioned, altruistic, altruism, sensible, courtesy |
| **Honesty and truth** | The quality of being sincere, transparent, and aligned with facts or reality. | 41 | honesty, honest, honestly, truth, truthful, true, truthfulness, the truth, real, realism |
| **Quantity, plurality, and size** | The amount, number, or extent of things or groups within a given context. | 41 | many, quantity, others, various, some, large, numerous, vast, giant, few |
| **Worry** | Unease about potential problems or risks. | 41 | worry, worrying, worried, concern, concerns, concern for people, uneasy, apprehension, worry about residents, worry about their inhabitants |
| **Energy** | The power derived from physical or natural resources to perform work or sustain activity. | 40 | energy, energy security, electricity, renewable energy, solar panels, windmills, clean energy, energy independence, energy conservation, power supply |
| **Guarantee** | A formal assurance or promise that certain conditions or outcomes will be met. | 40 | guarantee, guarantees, to guarantee, ensure, reassurance, reassuring, reassured, insure, assurance, grant guarantee |
| **Inequality** | The uneven distribution of opportunities, resources, or rights among individuals or groups. | 40 | inequality, social inequality, inequalities, unequal, financial inequalities, inequality in access, reduce inequalities, disparity, not the same for everyone, two-tier |
| **Culture and traditions** | The shared customs, beliefs, and practices that define a community or society. | 39 | culture, traditions, traditional, cultural heritage, values, tradition, heritage, multiculturalism, cultural integration, history |
| **Self and others** | Related to mentions of self and others. | 38 | me, he, them, us, self, we, myself, you, personal, ourselves |
| **Alliances and collaboration** | Partnerships and cooperative efforts between individuals, groups, or nations to achieve common goals. | 35 | alliances, strong alliances, support of an ally, alliance, collaboration, partners, coalition, pacts, partnership, allies |
| **Distrust** | Emotion (see Trust) - The absence of confidence or faith in the honesty or reliability of someone or something. | 35 | distrust, no trust, don't trust, lack of trust, mistrust, not trusted, untrustworthy, cannot be trusted, there is no trust in government, I don't trust |
| **Joy** | Emotion | 35 | joy, glad, happiness, happy, excited, upbeat |
| **Oppression and censorship** | The suppression of freedom or expression through control, restriction, or discrimination. | 35 | oppression, censorship, censored, repression, repressive measures, repress, persecution, suppressing opposition, oppressive, overbearing |
| **Sanctions and fines** | Penalties or financial charges imposed as punishment or to enforce compliance. | 35 | fine, fines, sanctions, sanction, heavy fines, increase fines, firmer sanctions, expanded sanctions, small fines, to collect fines |
| **Customs** | Traditional practices or habitual behaviours observed within a culture or community. | 34 | customs, customs administration, customs control, customs duty, customs policy, customs clearance, customs duties, customs officers, the customs, strengthen customs |
| **Enforcement - concept** | The application or execution of laws, regulations, or rules by authorised bodies. | 32 | enforcement, selective enforcement, enforce, lack of enforcement, harder enforcement, not enforced, no enforcement, low enforcement, ineffective enforcement, enforcement if necessary |
| **Training** | The process of developing knowledge, skills, or abilities through instruction and practice. | 32 | training, training in the event of a threat, training of uniformed services, best training, greater training, training actions, public training, better training, exercise |
| **Attention and inattention** | The focus or lack of focus directed towards something. | 31 | attention, attentive, inattention, constant attention, watch out, little attention, pay attention |
| **Cooperation** | The act of working together towards shared goals or mutual benefit. | 31 | cooperation, global cooperation, cooperation between security forces, cooperation between agencies |
| **Fauna and flora protection** | Efforts and policies aimed at conserving wildlife and plant species and preserving biodiversity. | 31 | animal welfare, animals, animal protection, cruelty to animals, protected species, wildlife conservation, flora and fauna protection, species protection (animals- plants), marine protection, animal rights policy |
| **President** | The head of an organisation - or the state. | 31 | President, Mr President, presidential protection, president's signature, protection on President of the State, change the president |
| **Transparency** | The quality of being open, clear, and accountable in actions, decisions, or communications. | 31 | transparency, transparent, transparency and accountability, non -transparent, clear, transparency in the media and government |
| **Agreements and appointments** | Formal arrangements or commitments between parties, often involving positions or responsibilities. | 30 | contract, contracts, appointments, agreements, agreement, agree, reaching out for agreements |
| **Biased** | Related to preference or prejudice in favour of or against something or someone unfairly. | 30 | bias, biased, favouritism, one-sided, stigmatizing, prejudices, prejudice, stigma, politically biased policy |
| **Commerce and trade** | The exchange of goods and services, both domestically and internationally, within economic systems. | 30 | tariffs, trade agreements, trade, trade protection, international trade, trade war, world trade, export, exchange, import quotas |
| **Minorities** | Groups distinguished by ethnicity, religion, or other characteristics that make up a smaller portion of a population. | 30 | minorities, minority, minority rights, minority protection, minority groups, protection of minorities, protect minorities, security for minorities, gypsy, rights for the Roma community |
| **Prohibition** | The act of formally forbidding an action or behaviour by law or authority. | 30 | prohibition, restricting, restriction, prohibitions, ban, restrictions, restrictive, not to allow, smoking bans, party bans |
| **Readiness and preparedness** | The state of being fully equipped and mentally or physically ready for a situation or task. | 30 | preparedness, prepping, readiness, ready, good preparedness, poor preparedness, ready yourself |
| **Protest, riot, and revolution** | Acts of public dissent, unrest, or radical change challenge established authority or systems. | 29 | right to protest, protests, protest, demonstration, riots, insurrection, revolution, strike, shout, confirmation of demonstration and protest |
| **Recruitment and reinforcement** | The process of enlisting new members or strengthening an existing group or force. | 29 | recruitment, recruitment needed, reinforcement, reserve recruitment, recruit more teachers, increase in enrolment, recruitment of police and army workers, recruiting the ultra -Orthodox |
| **Scarcity** | The limited availability of resources relative to demand or need. | 29 | scarce, who are scarce, scarce about |
| **Spaces and areas** | Physical or conceptual locations defined by boundaries or specific characteristics. | 29 | safe spaces, secure area, areas, area, local safety, safe place, city safety, protected places, safe places, on land |
| **Time** | Time-related concepts. | 29 | time, time-consuming, wait, current, now, when, long term, annual, ongoing, short term |
| **Belonging and connection** | Emotional need | 28 | contact, ties, network, social network, groups, belonging, relations, clans, membership, relationships |
| **Brands and companies** | Organisations or commercial entities that produce, market, or sell goods and services under a distinctive identity. | 28 | company, companies, brand, logo, slogans, Amazon (company), Google (company), Microsoft (company), Securitas (private security company), Saab (Swedish company) |
| **Safe daily life** | The condition in which individuals can live their everyday routines free from threats, harm, or insecurity. | 28 | safe neighbourhood, safe streets, street safety, security in the streets, more security on the streets, safety on the street, to be able to move safely, daily protection, walk the streets, security on streets |
| **Society** | A structured community of people bound together by shared institutions, culture, and relationships. | 28 | society, civil society, society security, responsible society, a well-functioning society, keeping society safe, societal safety, societies |
| **Species** | Groups of living organisms sharing common characteristics and capable of interbreeding. | 28 | wildlife, endangered species, flora, fauna, dog, dogs, cats, frogs, horses, wolf |
| **Terror** | Extreme fear or the deliberate use of fear to intimidate or control people. | 28 | terror, terror measures, terror fighting |
| **Benefits** | Advantages, assistance, or positive outcomes. | 27 | benefits, benefit, beneficial, social benefits, perks, compensation, allowance |
| **Fight and struggle** | Active effort or conflict aimed at overcoming challenges, resistance, or opposition. | 27 | fight, fighting, fighter, fighters, struggle, help in fighting, fight fire with fire, brawl, fight for the country, countering interference |
| **Governance and management** | The processes and structures through which organisations or states are directed, controlled, and held accountable. | 27 | governance, management, good management, good governance, mismanagement, good practices, ministers, lack of governance, bad management, board |
| **Keeping** | The act of maintaining, preserving, or retaining something in a particular state or condition. | 27 | keeping, keep, keeping on a regular basis |
| **Service** | Work or assistance provided to others. | 27 | service, services, activities of the services, service to a citizen, utilities, facilitated contact with services |
| **Gender and sexual orientation** | Aspects of identity relating to gender expression, roles, and individual patterns of sexual attraction. | 26 | gender rights, trans rights, LGBT, LGBT protection, LGBT rights, LGBTQ+, LGBTQ+ protection, LGBTQ+ rights, transgender, sexual orientation protection |
| **Nationality, origin and naturalisation** | The legal or cultural identification of individuals with a nation, and the process of acquiring citizenship. | 25 | nationality, national, naturalization, become a Swedish citizen, German, Swedes, native, Irish people, Czech nationality, fatherland |
| **Patriotism and nationalism** | Loyalty and devotion to one’s country, sometimes extending to the belief in its superiority or interests. | 25 | nationalism, national interests, patriot, patriotism, patriotic, best for Ireland, looking after the country’s interest, increasing patriotism, do more for your own country, protecting local goods |
| **Public security** | The safeguarding of citizens and property through collective measures and law enforcement activities. | 25 | public security, public security measures, efficient public security, safe in public, safety in public space, security in public space, public protection system, safe public spaces, protection of public places, public places protection |
| **Understanding and empathy** | The ability to comprehend others’ feelings, perspectives, and experiences with sensitivity and awareness. | 25 | understanding, understand, understandable, comprehension, empathy, empathetic, understood by everyone, understanding situation |
| **Careful** | Exercising attention, caution, or consideration to avoid mistakes or harm. | 24 | careful, be careful, meticulously, zealous, diligence |
| **Leadership and guidance** | The act of directing, influencing, and supporting individuals or groups toward goals or improvement. | 24 | leadership, strong leadership, leaders, guidance, guide, lead, strong leaders, team head |
| **Treatment** | Treatment-related terms. | 24 | treatment, mistreatment, treatment guarantee, providing treatment, I need treatment, treatments, recovery, protective treatment (legal/medical) |
| **Victims** | Terms related to Individuals who suffer harm, loss, or injury as a result of adverse circumstances. | 24 | victim, victim support, victim protection, protect victims, innocent victims, victimisation, help crime victims, support for victims of violence, support for violence victims, assistance to victims of attacks |
| **Emigration** | The act of leaving one’s country to settle permanently in another. | 23 | emigration, emigrant, emigration stop, emigrating, uncontrolled emigration, emigration from Israel, limited emigration |
| **Ethnicity** | A social category defined by shared cultural heritage, ancestry, language, or traditions. | 23 | ethnic, ethnicity, white (ethnicity), Muslims, Muslim, Arabs, race relations |
| **Indifference** | A lack of sympathy or concern towards the object or situation. | 23 | indifference, meh (indifference), apathy, indifference/apathy, political indifference |
| **Resources** | The assets, materials, or capacities available for use in achieving objectives. | 23 | resources, human resources, human capital, tools/aids, tool, resource, means, little means, increase resources for prevention, protection of material resources |
| **Bureaucracy** | A system of administration characterised by formal rules, procedures, and hierarchical organisation. | 22 | bureaucracy, less bureaucracy, too much bureaucracy, red tape, protection against the rule of bureaucrats |
| **Comfort** | A state of physical or emotional ease, free from pain or distress. | 22 | comfort, comfortable, mental comfort, convenience |
| **Deterrent** | Something that discourages or prevents certain actions, often through fear of consequences. | 22 | deterrent, nuclear deterrent, nuclear deterrents, police as a deterrent, deterrence, dissuade/deter, deterring / intimidating, better deterrents, dissuasive power |
| **Interest** | Curiosity or attention directed towards a person, topic, or activity. | 22 | interest, interests, interesting, best interest, more interest, interests of citizens |
| **Listening** | Listening and hearing terms. | 22 | listening, listen, listen to people, do not listen, lack of listening, more listening, heard, first I hear |
| **Reliability** | Terms related to reliability - quality of being dependable, consistent, and trustworthy. | 22 | reliability, reliable, product reliability, reliable timetable |
| **Awareness** | Conscious understanding or recognition of a situation, fact, or issue - individual or social. | 21 | awareness, collective awareness, creating public awareness, safety awareness, self-awareness, awareness of people, raise awareness, be security conscious, conscientiousness, sensitize |
| **Ethics and ideals** | Moral principles and values guiding behaviour and decision-making. | 21 | ideals, ethics, ethical, moral, principles, values, humanistic values, ethics protection, moral duty, code of conduct |
| **Evil** | Profound immorality or wickedness is regarded as a deliberate force or quality. | 21 | evil, cruelty, inhuman, bad intentions, bad people, unkind, vanity, monsters, incivility, malicious people |
| **Isolation and loneliness** | The state of being separated from others, physically or emotionally, leading to feelings of detachment. | 21 | isolation, loneliness, lonely, social exclusion, alone, secluded, never alone |
| **Negotiation** | The process of discussion aimed at reaching an agreement between parties with differing interests. | 21 | negotiation, negotiations, negotiating skills, good negotiations, compromise, compromises, proposal, concessions |
| **Neighbourhood** | Related to a local area or community where people live and interact socially, and the people who live there. | 21 | neighbours, neighbourhood, neighbours, neighbourly relations, secure neighbourhoods, neighbourhood groups |
| **Youth** | Related terms to youth: the period of life between childhood and adulthood. | 21 | youth, youth safety, youth centres, young, young people, more youth centres, protect youth, prioritising youth, against young people, protection for young people |
| **Delinquency** | Minor criminal or antisocial behaviour, especially among young people. | 20 | delinquency, delinquency prevention, eliminate delinquency, delinquent, delinquents, rampant delinquency, juveniles |
| **Dignity** | The state or quality of being worthy of respect, honour, or self-esteem. | 20 | dignity, human dignity, protection of human dignity, secure human dignity, ensuring dignity, honour, solemn |
| **Natural disasters** | Severe and sudden natural events, such as earthquakes or floods, causing widespread damage or loss. | 20 | natural disasters, protection against natural disasters, floods, flood protection, flood measures, natural disaster preparedness, earthquakes, storm protection, earthquake, storm forecast |
| **Palestine** | Related to Palestine | 20 | Gaza, Hamas, Palestinians, occupation, Judea and Samaria, support for Gaza's oppressed children, Hamas elimination, blow up Gaza, burn Gaza, take away their land |
| **Targeted** | Specifically directed at an individual or group for attention, action, or attack. | 20 | targeted, target |
| **Visibility** | The degree to which something or someone can be seen, noticed, or recognised publicly. | 20 | visible, visibility, observation, observe, to watch, watching, look, see, seen, eye-catching |
| **Civil** | Relating to citizens and their rights, duties, or social relations within a community or state. | 19 | civil, civility, civil servants, civil service |
| **Crisis** | A time of intense difficulty, danger, or instability requiring urgent action or resolution. | 19 | crisis, crisis plan, crisis staff, crisis management, crisis knowledge, anti -crisis, preparedness and crisis management, calamity, calamity protection, crisis telephone lines |
| **Disappointment** | Emotion | 19 | disappointment, disappointed, disappointing |
| **Impossibilities and utopias** | Ideas, situations, or goals that are unrealistic or represent idealised perfection beyond reach. | 19 | impossible, inapplicable, impotence, inability to influence, lack of influence, impasse (deadlock), utopia, cannot, liability, non-exit |
| **Physical safety** | The condition of being protected from bodily harm or physical danger. | 19 | physical protection, physical security, physical, physical safety, physical and material integrity, protect people physically |
| **Protection against racism** | Measures or issues related to safeguarding individuals from racial discrimination and prejudice. | 19 | protection against racism, anti-racist, anti-racism policies, the fight against racism, no racism, free from racism, racial equality, not racist, ban on racial attacks, BLM (Black Lives Matter movement) |
| **Reform** | The process of making changes to improve systems, laws, or institutions. | 19 | reform, reformer, a legal reform, social aid reforms, fair reforms, reformed, adequate reforms, daycare reform |
| **Security services and system** | Organisations and mechanisms responsible for the protection of people, property, and information. | 19 | security services, security service, the security system, law enforcement services, a strong security system, general security service, safety services, providing security products |
| **Abuse and abuse prevention** | Harmful treatment of others and the actions taken to stop or prevent such behaviour. | 18 | abuse, abusive, responding to abuse, abuse prevention, anti-abuse, protection from abuse |
| **Availability and accessibility** | The extent to which something is obtainable, usable, or reachable by those who need it. | 18 | availability, accessible, accessibility, access, have access to, availability of units, availability of the health facilities, available transport, keys |
| **Bombs and explosions** | Devices or events causing destructive blasts, often associated with warfare or terrorism. | 18 | bombing, bombers, bombs, bombings, bomb squad, bomb, explosions, explosion, explosive |
| **Bunker and fortress** | Strongly built shelters or defensive structures designed to protect people or assets from attack or disaster. | 18 | bunker, bunkers, protective bunker, secure bunkers, build a bunker, fortress |
| **Business** | The activity of producing, buying, or selling goods and services for profit. | 18 | business, business security, security for businesses, safeguard businesses from harm, enterprise, corporations, stakeholders |
| **Confidence negative** | The lack of trust or belief in oneself, others, or a system. | 18 | no confidence, absence of confidence, there is no confidence, there is not enough confidence, too little confidence in women, a shaky confidence, lack of confidence, and growth |
| **Enemies** | Individuals, groups, or nations regarded as hostile or opposed. | 18 | enemies, enemy, eliminate the enemies, protection from enemies, we are surrounded by enemies, eradicate the enemies, the enemy destruction, against enemy, assault of enemy states |
| **Immunity** | Protection or resistance against disease, harm, or legal responsibility. | 18 | immunity, impunity, inviolability, immunized |
| **Measures** | Actions or steps taken to achieve a specific goal or address a situation. | 18 | measures, stricter measures, social measures, measure, an extraordinary measure, no further measures |
| **Opinion and advice** | Personal views or guidance offered to inform decisions or perspectives. | 18 | opinion, opinions, ask people’s opinions, world opinion, perspectives, advice, views, free opinion |
| **Security organisations, branches, and agents** | Institutions and personnel responsible for intelligence, surveillance, and protection operations. | 18 | security companies, security company, security firms, security agents, security bodies, security agency, security organizations, PSG Security, Verisure (security company), Prosegur (private security company) |
| **Ukraine** | Related to Ukraine | 18 | Ukraine, Ukrainians, Ukraine support, support for Ukraine, help for Ukraine, end of support for Ukraine, not to support Ukraine, remove Ukraine from Poland |
| **Abstract and conceptual** | Ideas or notions that exist in thought rather than in physical or concrete form. | 17 | concept, abstract, abstraction, idea, ideas, concepts, theory, incorrect concept |
| **Alcohol and drugs** | Substances that alter mood, perception, or behaviour are often regulated due to health and social effects. | 17 | drug-free, alcohol, alcohol ban, stricter retirement control for drugs, prohibition of driving under the influence of drugs, the availability of drugs, alcoholics, drug policy is too lax, no alcohol sales under 18, drug management, better control of drug misuse, anti-drug, cheaper drugs, beer from age 18, limit sales to already intoxicated people, needle exchange, marijuana legalization, detox centre, smoking ban, not addicted |
| **Broad coverage** | Coverage-related | 17 | covered, coverage measures, cover, coverage, covered, broad coverage, cover, coverage, covers |
| **Courage** | The ability to confront fear, danger, or adversity with strength and determination. | 17 | civil courage, courage, courage, bravery, put on a brave face, boldness |
| **Decision and preference** | The act of choosing between options based on priorities or desires. | 17 | decision -making, no decision -making, decisions, decisions, decision, right decision, decision, hard decisions, choices, resolutions, preference, selective |
| **Fast and speed** | The quality of moving or operating quickly or immediately. | 17 | fast, fast, faster, fast response to intruder, faster intervention, speed, quick, rapid action, immediate interventions, speed, 2 speeds, faster judgments, blitz |
| **Games, sports, and results** | Competitive activities and their outcomes or scores. | 17 | sport, the protection of the game, game, game, baseball bat, victory, victory, we will win, run, the game of life, ball, successes, do not retreat, play |
| **Home safety** | Practices and measures to protect people and property within domestic environments. | 17 | home security, home safely, home safety, safe homes, we’re not safe in our homes, personal security from home, security in the home, security at home, safety houses, house security, safe haven, in every home, close the houses, my home, on the door, domestic |
| **Humanity and humanitarian** | The collective human race and humanitarian-related. | 17 | humanity, humanity, human, fellow humans, fellow human, humanist, civilization, humanitarian |
| **Self-sufficiency** | The ability to provide for one’s own needs without external assistance. | 17 | self -sufficiency, 3 days of self-sufficiency, self-sufficiency, self-sufficiency, survival, self-protection, up to oneself, self -minded, if I am not for myself… |
| **Silence** | The absence of sound or speech often implies calm, respect, or suppression. | 17 | quiet, quiet, silence, live in quiet, quiet in the streets, law of silence, silence |
| **Threat - radicalisation and extremism** | The danger posed by individuals or groups adopting extreme beliefs leading to potential violence or unrest. | 17 | radicalisation, right -wing extremism, extremists, radicalism, action plan against right -wing extremism, left extreme, far-right extremism, radical Islamism, right -wing extremist, polarization between groups, resistance to right -wing extremism, no extremism, prevent duty (UK anti-extremism programme), nazi, extremely |
| **Tranquillity** | Free from disturbance or stress. | 17 | tranquillity, tranquillity, tranquillity to get around, generate tranquillity |
| **Belief** | Acceptance that something exists or is true, often without complete proof. | 16 | belief, belief, faith, faith in institutions, faith, faithful, faith protects, believed |
| **Characteristics** | Distinguishing traits or qualities that define a person, object, or concept. | 16 | characteristics, popular, type, open, authentic, polishness, flexibility, obedience, curiosity, curious, tough, secular (as adjective), simple |
| **Conflicts and disagreements** | Situations where opposing views or interests lead to tension or dispute. | 16 | many confrontations / many clashes (literal: “many eye conflicts”), conflict, bickering, internecine (internal conflict), disagreement, disagreements, anti, opposition, dispute, quarrels, discord/disagreements |
| **Discipline** | Controlled behaviour and adherence to rules or training. | 16 | disciplined, discipline |
| **Distances** | The measure or perception of space between points, people, or ideas. | 16 | 1-meter distance, 2-meter distance, distance, keep a distance, distance, proximity, far, near |
| **Fearless** | Absence of fear. | 16 | freedom from fear, live without fear, no fear, not create fear, fearless, lack of fear, not to be afraid, don't be afraid, unafraid to express oneself, the people are not afraid of the long way, do not be afraid of the lying un |
| **Future** | Related to the future. | 16 | future, future (as in future-oriented), a future, there is no future, future-proof, after, will be realized, afterwards, forecast |
| **Hygiene** | Practices that maintain health and prevent disease through cleanliness and sanitation. | 16 | hygiene, hygiene, hygiene gels, hygiene regulation, functional sanitation, sanitary, sanitary prevention, shampoo, wash your hands, sanitize, clean street |
| **Manipulation** | The act of influencing or controlling others, often deceptively or unfairly. | 16 | manipulation, manipulation (typo), manipulation, manipulations, social democratic spin, indoctrination of citizens, manipulators, manoeuvre, lure, scheming, pretend |
| **Minors** | Individuals under the age of legal adulthood. | 16 | better supervise minor delinquents, internet restriction for minors, minors, protection of minors, minors, minor, minors alone |
| **Night** | Night-related | 16 | night, nightlife, at night, night, safe nightlife, security night, night shifts (likely typo), more security in the night, we are not safe to walk the streets day or night, walking alone at night is a no-go, evenings |
| **Not understanding** | Lack of comprehension | 16 | I don't understand. I did not understand, I don't understand the question, I don't understand. I don't quite understand the question, I do not understand you, difficult to understand, I don't understand you, I don't understand the question, I don't understand |
| **Budget** | A financial plan outlining income, expenses, and resource allocation over a period. | 15 | funds, augment the budget, a large security budget, budget the security system, underfunded, budgetary, security budget, a high priority security budget, lack of budget, investments, need more funding, need a budget to all this, investment required |
| **Disinterest** | A lack of enthusiasm, engagement, or concern about something. | 15 | disinterest, disinterested, don't need protection, uninteresting, boring, i do not need, no need, unnecessary (corrected typo), not relevant, overhyped, irrelevant, unnecessary thing, indifferent, is not used for much, secondary, no interest, do not care, busy with more important matters, we do not need them really, disinterest, disinterest- indifference, uninteresting, little interest, not needed, dispassionate, doesn't matter,... I don't care, don't care, coughs / doesn't care (context-dependent), I don't care, nullity, indifferent |
| **Goods and essential goods** | Tangible products, especially those necessary for basic living needs. | 15 | access to essential goods, ceilings for the prices of essential goods, protection of goods, protection of people's goods, security of goods, products, protection of belongings, protection of objects, asset protection, Polish product, security of assets, goods |
| **Presence** | The state of being in a place or situation, physically or symbolically. | 15 | presence, presence, present, presence on the street, greater presence of safety force, distinct presence, show up |
| **Reserve and reservation** | The act of setting something aside for future use or the arrangement of securing access in advance. | 15 | reserve, reserved, active reserves, reservations, reservation, tangible reserves, reserve |
| **Serenity** | A state of inner peace, free from agitation. | 15 | serenity, serenity, serene |
| **Anxiety** | An emotional/physical worry or unease about uncertain outcomes. | 14 | anxiety, anxiety about defence, anxiety, anxious, nervous |
| **Disgust** | Emotion | 14 | disgust, disgusting, nausea |
| **Helplessness** | The feeling or condition of being unable to act or control a situation. | 14 | helplessness, there is no help from the state, helpless, unhelpful, no help, we have become helpless, do they help?, lack of help |
| **Love** | A deep affection, care, or emotional attachment towards someone or something. | 14 | love, loved, lovely |
| **Identity - general** | The collective aspects that define who a person or group is. | 14 | protected identity, identity, safe identity, identity, protected identities, identification, DNA, fingerprints |
| **Nuclear** | Relating to atomic energy, weapons, or reactions, often associated with power or defence. | 14 | nuclear threat removal, nuclear weapons, nuclear weapons (corrected typo), nuclear weapons - terrible!, radioactivity protection, warning about RCB threats, no-fly zone over Temelín (nuclear plant), atomic attack warning, denuclearization, nuclear security, nuclear |
| **Protection against danger and risk** | Safeguarding from potential harm, threat, or exposure to hazardous conditions. | 14 | prevention of danger, no danger, eliminating danger, risks protection, risk management, protection from personal safety risks, reduce risk, lower risks, low risk, protect people at risk, buffer, protection against an aggressor, without danger |
| **Selfish and self-centred** | Concerned primarily with one’s own interests or needs, often disregarding others. | 14 | selfishness, ego, narcissism, self-centred, egoism, each for itself, egoists, they think about themselves, they only worry about their seat/position, self -realization at the expense of others |
| **Against violence** | Opposition to physical aggression or harm as a principle or policy. | 13 | fight against violence, against violence, fight against violence on the part of left -wing extremists, combat violence, counteracting violence, measures against violence, avoid conflicts, conflict-averse, package of measures against violence, non-aggressive, no retaliation, 1522 (Italy’s anti-violence helpline) |
| **Curfew** | A regulation requiring people to remain indoors during specified hours. | 13 | curfew, curfews |
| **Death** | The end of life: the irreversible cessation of all vital functions. | 13 | death, immortality, death at work, funeral, more support after loss, right to die |
| **Locks and locking** | Mechanisms or actions used to secure entry or restrict access. | 13 | locks, lock, locking up, lock up, lock my door in daytime, locked, security locks, the triple lock, lock |
| **Privileges** | Special rights, advantages, or immunities granted to particular individuals or groups. | 13 | privileges, privilege, privileges/exemptions, confidentiality/privilege, for the chosen ones, protection of the rich, keeping the rich safe, rich people, |
| **Body** | The physical structure of a person or living organism. | 12 | hair, buttocks/ass, head, butt, bone, heart, hands, bodily, skin (genitive), shoulder, shoulders, blood |
| **Clothing** | Garments and attire worn for protection, modesty, or expression. | 12 | wear coloured clothing, scarf, gloves, coat, clothes, uniformed, in the jacket, uniformed services, suit, standards of uniformed services, cape, burqa |
| **Crown** | Crown and monarchy related. | 12 | king (monarch), royal family (Norwegian/Danish form), king (royalty), Kate (personal name or royal figure), royal house, the king (royalty), royal (monarchy or noble), crown, king, king, a legal monarchy |
| **Disarm and seizure** | The removal or confiscation of weapons or dangerous materials. | 12 | disarm, seizure of an item, disarmament, do not arm, disarmament based on the past, confiscation of items, disarmament, seizure, unarmed, confiscate luxury items |
| **Enlightenment** | The state of gaining knowledge, insight, or understanding; historically, a philosophical movement promoting reason. | 12 | enlightenment, enlightened, enlightenment, to be enlightened- strong- and advanced |
| **Men** | Adult human males are often discussed in relation to gender roles and social dynamics. | 12 | male culture, men, male-led (descriptive term), man, guys, very man, men |
| **Neutrality** | The state of not taking sides in conflict or controversy. | 12 | neutrality, neutral, neutrality, Ireland’s neutrality |
| **NGOs and other organisations** | Non-governmental and civil entities working for social, humanitarian, or advocacy purposes. | 12 | Santa Casa da Misericórdia (charitable institution), Caritas (catholic charity), Casa Pia, foundations for terminally ill patients, NGOs,foundations, *die tafeln* (charity institutions), care companies, white circle of safety |
| **Nobody** | No person; the absence of any individual or identity in a context. | 12 | nobody |
| **Preservation and conservation** | The protection and maintenance of natural, cultural, or material resources to prevent loss or decay. | 12 | preservation, preserve, conservation, preserve |
| **Propaganda and publicity** | Information spread to influence opinion or promote a cause, often with biased intent. | 12 | propaganda, publicity, marketing, advertising |
| **Regulatory** | Related to the establishment and enforcement of rules or standards. | 12 | regulatory office, regulatory authorities, regulatory bodies, regulators, regulatory funds |
| **Retirement** | The withdrawal from active employment is typically after reaching a certain age. | 12 | retirement, retirement age, retired |
| **Shame** | Emotion | 12 | shame, shame, shameful, shameless, ashamed |
| **Cheating** | Acting dishonestly to gain an unfair advantage. | 11 | cheating, cheating, cheated, cheaters, cheater, cheater, treason, treason, traitor |
| **Holistic and general** | Holistic and general terms | 11 | universal, general, a holistic approach, touches everyone, multi-front / multi-sector, generally, encompassing, without being seen in a whole |
| **Hope** | Emotion | 11 | hope, hope (idiom), hope nothing happens, hopeful |
| **Organisation** | A structured group of people working together to achieve specific goals. | 11 | organisation, organisations, better organisation, establishment |
| **Staff** | The group of people employed by an organisation. | 11 | staff, short-staffed, more staff, too few staff, greater staffing, staff, will staff need to participate, personnel, additional personnel are required |
| **Strategy** | A planned approach designed to achieve specific objectives or overcome challenges. | 11 | strategy, strategy, serious security at key sites, strengthening the weaker layers |
| **Threat - disinformation** | Spread of false or misleading information. | 11 | defence of hybrid threats and disinformation, fight with misinformation, disinformation, false information, whataboutism is spreading, combating disinformation, children exposed to dangerous ideology in schools |
| **Type of activity** | type of activity or of the person doing the activity | 11 | active, proactive, reactive, passive, reactivity, security activities, hyperactive, activity, interactive |
| **Block and remove** | The act of obstructing access or eliminating something undesirable. | 10 | block, delete, stop, stop, block, do not, get rid of something, abolish/remove these associations, halt |
| **Development and construction** | The process of growth, improvement, and building of infrastructure or systems. | 10 | development, caring for development, construction/development, reconstruction, reconstruct, develop |
| **Evacuation** | The organised removal of people from a dangerous or threatened area. | 10 | evacuation, evacuation and emergency plans, evacuation, red zone evacuation plan, evacuation of residents, evacuated |
| **Good feeling** | A sense of positivity, satisfaction, or emotional comfort. | 10 | good feeling, goodness, life is good, people felt good, positivity, I'm doing fine, to feel that there is someone looking out for me, animation, smile, not humiliation |
| **Industry** | Economic activity is concerned with the processing of raw materials and the manufacturing of goods. | 10 | industry, building industry, own arms industry, higher expenditure of the arms industry, industrialists, relieve industry |
| **Integrity** | The quality of being honest and having strong moral principles. | 10 | integrity, integrity |
| **Jews and antisemitism** | References to Jewish people and prejudice or hostility directed against them. | 10 | Jewish attacks, demolishing houses of Jews, jews will always have to protect themselves, jews, the control of the Jews, strategy against anti-Semitism and for Jewish life, against anti-Semitism, anti-Semitism, Jews are brothers, the people shall dwell alone |
| **Mission, vision, and programmes** | The purpose, guiding goals, and planned initiatives of an organisation. | 10 | mission, mission, missions, programs, program, vision, recording programs |
| **Names** | Words or terms used to identify people, places, or things. | 10 | Manuel (name), Alana (first name), Brigitte (given name), Franek (male name), Roman (male name), Robert (name), Elisabeth (given name), Jake, Bob |
| **Permission** | Authorisation or consent to do something. | 10 | security clearance, strong clearance, permission, council permissions, permission, consent, consent, permissive |
| **Principles of sovereignty** | The fundamental rights of states to govern themselves without external interference. | 10 | sovereignty, national sovereignty, legitimacy, legitimate, sovereign rights, self-determination |
| **Promises** | Commitments or assurances that something will be done or will happen. | 10 | keep promises, promise, promises, they promise too much, make promises |
| **Seriousness** | The quality of being sincere, important, or demanding careful consideration. | 10 | seriousness, seriously, take seriously, serious |
| **Supplies and stocks** | Essential materials or resources, kept for use or emergency needs. | 10 | stock, supply, sources of supply, supplier |
| **Clarity** | The quality of being clear, understandable, and free from confusion or ambiguity. | 9 | clarity, clarity, not ambiguous, clear plan, the clear position, clearly |
| **Close** | To shut, end, or bring something to completion; also denotes proximity. | 9 | closing, close, closed, closeness, close, closure, closures, closure |
| **Defeat - general** | The experience of loss or failure in conflict, competition, or personal endeavour. | 9 | defeat, withdrawal, desist, step back / retreat, giving up, surrender, submission |
| **Directions and reach** | Words and expressions related to directions and reach | 9 | upward, up, forward, overreaching, high, high, next, direction, upper, down |
| **Dissatisfaction** | A sense of unhappiness or frustration caused by unmet expectations. | 9 | unsatisfactory, dissatisfaction, unsatisfactory, not the most satisfied |
| **Infractions and violations** | Acts of breaking laws, rules, or ethical standards. | 9 | violation, do not violate the rules, preserve against violation, infractions, charges, involvement of offenders' families, copyright infringement, disobedience |
| **Living conditions - general** | The circumstances affecting people's living conditions in general. | 9 | a living, living in the right area, protection of living standards, a safe standard of living, maintaining the right standard of living, expansion of living opportunities, living conditions, standard of living, basic amenities |
| **Maternity protection** | Maternity and measures ensuring the health, rights, and employment security of pregnant women and mothers. | 9 | better maternity terms, maternity, maternity benefit, maternity leave, maternity protection, maternity, protection of single mothers |
| **Negativity** | A pessimistic or critical attitude that emphasises adverse aspects. | 9 | pessimistic, depreciation, negativity, pessimism, sceptic, pessimism, discomfort |
| **Obligation** | A duty or commitment to perform an action or uphold a responsibility. | 9 | obligations, obligation, obligations, mandatory, mandatory for a citizen, we have no other option, I have to |
| **Resident** | A person who lives permanently or for an extended period in a particular place. | 9 | resident, residents, residences, reside, residence |
| **Sadness** | Emotion | 9 | sadness, sad, sad, unhappy, unhappy, unhappy protection |
| **Segregation and marginalisation** | The separation or exclusion of individuals or groups from mainstream society or opportunities. | 9 | exclusion (typo), segregation, ghetto, excluded, marginalization, marginalized |
| **Separation and division** | The act or state of being split apart, whether physically, socially, or ideologically. | 9 | division, separation, split/division, the people of Israel are divided, divided, divisive, factions |
| **Severity** | The degree of seriousness or intensity of a situation, condition, or punishment. | 9 | severity, severe, severe, severity |
| **Sex and sexuality** | Biological and social aspects of sexual identity, attraction, and behaviour. | 9 | condoms, sexual, sexuality, sexuality, sex, sexual protection, contraception, sex in prisons |
| **Traumatic events** | Events documented for causing severe emotional or psychological distress. | 9 | October 7, holocaust, seventh in October, October 7, October |
| **Unrest** | A state of dissatisfaction or disturbance within a society, often leading to protest or disorder. | 9 | unrest, a restlessness, restlessness, world unrest |
| **Waste and garbage** | Unwanted or discarded materials resulting from human activity. | 9 | waste, waste sorting, sorted waste, garbage cleanup, protection against waste, waste resources, trash/garbage |
| **Acceptance** | The act of agreeing to or recognising something or someone as valid or true. | 8 | acceptance, acceptance despite race, acceptance is needed, acceptance of the other, accept, socially acceptable behaviour |
| **Age** | Age | 8 | age, age-related services, ages, all ages |
| **Arrogance** | An attitude of superiority and excessive self-importance. | 8 | arrogant, arrogance, arrogance, condescension, arrogant |
| **Autonomy and independence** | The ability to act or make decisions freely without external control. | 8 | autonomy, give autonomy, independent, independence, independent, independence |
| **Cleaning** | Cleaning | 8 | cleanliness, cleanliness, clean, clean, cleaning of spaces, cleaning |
| **Earth** | Planet Earth directly related words and expressions. | 8 | earth, earth protection, world, world, planet protection |
| **Hate** | Intense dislike or hostility towards someone or something. | 8 | hate, I hate, hate against Swedes, hatred, hate Irish, hate |
| **Intention and will** | Purpose or determination in acting. | 8 | willingness, will, intention, will, the intention, involuntary |
| **Intermediate and mediation** | The role or process of acting between parties to facilitate understanding or agreement. | 8 | mediation, mediators, mediate security, disregard for intermediary organisations, conflict resolution, calming the situation from time to time, intermediate |
| **Invasion and intrusion** | The act of entering a space, territory, or situation without permission or welcome. | 8 | invasive, invasion, anti-intrusion, infiltration, penetration |
| **Leak and escape** | Related to leak and escape | 8 | leak, leaky, escape, escape, evasive, evasion, exit |
| **Outside outdoors** | Areas or activities taking place in the open air rather than indoors. | 8 | aware of outside issues, the outside world, outside, from outside of Germany, out, don’t dare go outside, safe outside, outdoor spaces |
| **Problem** | A matter or situation regarded as harmful, difficult, or in need of resolution. | 8 | problems, problem, dk problem, problems, social problem, trouble |
| **Relief** | The alleviation of distress, discomfort, or suffering. | 8 | relief |
| **Support and benefits criticism** | Negative evaluation or disapproval of aid. | 8 | hard to claim (benefits), poor support, inefficient social security, false political promises of support, lack of support, lack of support for the trans community, there is no support |
| **Threat – hate speech** | Expressions intended to incite violence or hostility based on prejudice or discrimination. | 8 | hate speech, protection from hate speech, online hate, hate speech bullshit |
| **Unaware and unexpected** | Conditions or events that occur without prior knowledge or anticipation. | 8 | unaware, not aware of them, unexpected, not aware, unheard of |
| **Advocacy** | The act of supporting or arguing for a cause, policy, or group. | 7 | advocacy, advocacy, advocate |
| **Agriculture** | The practice of cultivating land and raising livestock for food and resources. | 7 | good agriculture, agriculture, green number, agricultural (corrected), IGP (protected geographical indication), farmland, fertilizer limits |
| **Anonymous** | Without a name or identifiable information, lacking recognition or identity. | 7 | an anonymous focus, anonymous, anonymity, anonymity |
| **Association** | An organised group of people united by a common purpose or interest. | 7 | associations, association, association, associations |
| **Balance** | A state of stability or harmony between opposing elements or forces. | 7 | balance, balanced, balanced |
| **Consequences** | The outcomes or results that follow from actions or events. | 7 | consequences, consequence, no consequences, dare |
| **Daily life general** | Routine activities and experiences forming part of ordinary existence. | 7 | daily effort, general safety when out day-to-day, safe to walk around, routine, in the park, going around / walking around, trying to get by |
| **Easy** | Easy | 7 | easy, ease, easy, ease |
| **Events** | Occurrences or happenings, especially those of significance or public interest. | 7 | past events, events, protect folk festivals, protection during major events, security at public festivals, more security at street festivals, cancellation of the celebrations |
| **Hostages** | Individuals held captive to influence negotiations or outcomes. | 7 | release hostages, bring back the hostages at any cost, to bring back hostages, giving up on hostages, returning the hostages, return of abductees/hostages, captivity |
| **Meet** | To come together or encounter someone, often for discussion or exchange. | 7 | meeting, meetings, meeting, meet, cabinet meetings, conference, check meetings |
| **Radar** | A detection system using radio waves to identify the presence and distance of objects. | 7 | radar controller, radars, individual radars, radar, radar measurements |
| **Secret** | Information kept hidden or known only to a limited group. | 7 | secretive, secret, secret, secrecy, mystery |
| **Standard** | A level of quality or performance regarded as a norm or requirement. | 7 | standard, standards, standard, standards (misspelled) |
| **Strangers** | People who are unknown or unfamiliar to one another. | 7 | don’t talk to strangers, stranger, strangers, stranger |
| **Sustainability** | The practice of meeting current needs without compromising the ability of future generations to meet theirs. | 7 | sustainability, sustainability, sustainable development, sustain |
| **System** | An organised structure or set of processes working together for a specific purpose. | 7 | system, systems, fair system |
| **Accountability** | The obligation to explain and take responsibility for actions or decisions. | 6 | accountability, held accountable, hold perpetrators of violence accountable |
| **Colour** | The visual property derived from light reflection, often associated with perception and diversity. | 6 | colour, colour, white, dark, white |
| **Come** | related to the word "come." | 6 | comes, come, comes, come on |
| **Deprivation** | The lack of necessities or conditions essential for well-being. | 6 | deprivation, deprivation of liberty, deprive people, deprived suburbs |
| **Desire** | A strong feeling of wanting to have or achieve something. | 6 | desire, a desire to protect, it is to be desired, a desire for dictatorship, desirable |
| **Disbelief** | The refusal or inability to accept something as true or real. | 6 | disbelief, don't believe, I do not believe it, fantasy, I don't believe they are impartial, incredulous, there is no horizon |
| **Embarrassment** | A feeling of self-consciousness or discomfort arising from awkward situations. | 6 | embarrassing, embarrassed, embarrassing, embarrassment |
| **Hide and avoid** | To conceal oneself or something and steer clear of contact, exposure, or responsibility. | 6 | avoid, hide valuables, hidden things, hidden, concealed, avoid disorder, cop-out (colloquial term) |
| **Hopelessness** | Absence of hope. | 6 | hopelessness, hopeless, they are hopeless |
| **Inconsistency** | The state of lacking uniformity, coherence, or stability. | 6 | inconsistency, inconsistency |
| **Modern** | Characterised by contemporary ideas, methods, or technology. | 6 | modernization, modern, modernism |
| **Pain** | Physical or emotional suffering or discomfort. | 6 | big pain, pain, longer and direct pain, injury, bruised |
| **Populism** | Political approaches that appeal to ordinary people who feel overlooked by elites. | 6 | crawling populism, populism, populism, populist |
| **Prosperity** | A state of success or wealth, often involving economic and social well-being. | 6 | prosperity, prosperity |
| **Public** | Relating to the general population or open access for all members of society. | 6 | public, general public |
| **Public services** | Services provided by the government for the benefit of citizens. | 6 | public service, free public services, public services, develop public services, citizens' information (Irish public service), guarantee public services |
| **Resilience and determination** | The capacity to recover from difficulties and maintain commitment to goals. | 6 | resilience, endures, determination, sturdiness, don’t surrender to the dictates of the world |
| **Status** | A person’s social or professional standing or recognised position. | 6 | social status, status |
| **Tolerance-zero** | A strict policy of non-acceptance towards specific actions or behaviours. | 6 | zero tolerance, zero tolerance strategy, zero tolerance for drugs |
| **Unbiased** | Showing fairness and neutrality without prejudice or favouritism. | 6 | no bias or favour, unbiased, impartiality, impartial |
| **Underestimate** | To judge something or someone as less important or capable than they truly are. | 6 | underestimated, underestimate, underestimating danger, underestimation, underestimated |
| **Accident** | An unexpected and unplanned event causing damage or injury. | 5 | accident, accidents, accident-free, accidents |
| **Average** | Average | 5 | average, average, average |
| **Contributions and recommendations** | Inputs, suggestions, or proposals offered to support improvement or decision-making. | 5 | contributions, contributions, contribute, I recommend |
| **Coordination and organising** | The management and arrangement of people or activities to achieve efficiency and order. | 5 | coordination, synchronization, set in orders |
| **Effort** | The physical or mental energy exerted to accomplish something. | 5 | efforts, effort, effort, big effort |
| **Freedom - low** | A reduced state of liberty or personal autonomy due to restrictions or control. | 5 | lack of freedom, drop in freedom, low freedom, lack of freedom, no free speech |
| **Greed** | An excessive desire for wealth, power, or possessions. | 5 | greed, greed |
| **Heat** | The quality of being hot; energy associated with temperature or warmth. | 5 | warmth, heat, heating |
| **Needs - general** | Basic requirements essential for survival, comfort, or functioning. | 5 | you need, personal need, needs and wants, needs, unmet needs |
| **No answer** | Refusal to answer. | 5 | pass, can't say |
| **Non-state actors** | Individuals or groups that operate independently of government authority often influence political or social outcomes. | 5 | charities, some charitable foundations |
| **Performance** | The execution or accomplishment of a task, role, or duty. | 5 | performers, performative, perform, performance |
| **Process** | A series of actions or steps taken to achieve a particular outcome. | 5 | processed, processing time, processes, process |
| **Social justice** | The fair and equal distribution of rights, opportunities, and resources within society. | 5 | social justice, investment in social issues, every German should be treated fairly |
| **Structures** | The physical or organisational frameworks that support systems or activities. | 5 | structures, defensive structures, structure |
| **Travel and holidays** | The movement of people for leisure, business, or exploration, often involving time away from home. | 5 | travel, hotels, holiday, trips |
| **Volunteering** | The act of offering time and service freely to benefit others or a cause. | 5 | volunteer, volunteering, volunteers |
| **Abroad** | In or to a foreign country. | 4 | abroad, exhilarate abroad, abroad, support from abroad |
| **Airport security** | Measures and procedures ensuring the safety of passengers and staff at airports. | 4 | airport security, airport, more control at airports, insurer at airports, airport control |
| **Anger** | Emotion | 4 | anger, anger, I am angry with politics |
| **Celebrities** | Famous individuals known for their achievements or public recognition. | 4 | celebrities, protection of important people in the state, entourage |
| **Concrete and measurable** | Defined and quantifiable, capable of being assessed objectively. | 4 | measurement, measurable, and concrete actions, it must be concrete |
| **Condition** | The state or circumstances of something or someone at a particular time. | 4 | conditions, conditions, conditions of protection |
| **Influence** | The power to affect behaviour, development, or thinking. | 4 | influence, lobbyism, lobbying group, lobbying, convince |
| **Martial arts and self-defence** | Disciplines and techniques used for combat and personal protection. | 4 | martial arts in schools, self-defence course, free self -defence courses for children, right to self-protect |
| **Minimum** | Minimum | 4 | minimum, minimums, minimum, minimum level, the minimum |
| **Misery** | A state of great suffering, distress, or unhappiness. | 4 | miserable, miserable, misery |
| **Negative events** | Incidents or circumstances resulting in negative outcomes. | 4 | something not good happens, protection of mass events, cancel a public event, unfavourable events |
| **Panic** | Sudden uncontrollable fear or anxiety, often leading to irrational behaviour. | 4 | panic, panic -inducing, panic |
| **Provision** | The act of supplying or making something available for use. | 4 | providence, providing, provide, provision for all |
| **Recognition and appreciation** | Emotional need | 4 | appreciate those who protect me, feeling valued in home country, recognised, reputation |
| **Satisfaction** | Emotion | 4 | satisfaction, satisfaction |
| **Softness** | The quality of being gentle, mild, or easily shaped or affected. | 4 | softness |
| **Subcultures** | Groups within a society that develop distinct norms, values, or interests. | 4 | woke, ""trust me"" culture (or ""culture of reliance"")." |
| **Agenda** | A list or plan of items to be discussed or acted upon; also, a broader set of underlying intentions or priorities. | 3 | agendas, agenda |
| **Content** | The information, material, or ideas contained within something, such as a text or medium. | 3 | content, contents |
| **Decay and destruction** | The process of deterioration or damage leading to disintegration or ruin. | 3 | degradation of the cities, protection against social decay, eliminate non -rights areas |
| **Depression** | A state of persistent sadness or a mental health condition marked by low mood and loss of interest. | 3 | depression, depression forces |
| **Everyone** | All people without exception; every person collectively. | 3 | all, everyone, everyone |
| **Everything** | All things collectively; the entirety of what exists or is considered. | 3 | do everything, everything, that's all |
| **Experience** | The knowledge or skill gained through involvement in or exposure to events. | 3 | experience, experiencedly, experience |
| **Facts** | Objective realities or pieces of information that can be proven to be true. | 3 | fact, facts, facts |
| **Follow** | To go or come after something; to adhere to or observe a rule, idea, or person. | 3 | follow indications, followed, follow |
| **Gratitude** | Emotion | 3 | grateful, gratitude, gratitude |
| **Guilt** | Emotion | 3 | guilt, remorse, a guilty conscience |
| **Harmony** | A state of balance and agreement among people, ideas, or elements. | 3 | harmony |
| **Instability** | The condition of being unsteady, unpredictable, or prone to change or collapse. | 3 | instability |
| **Office** | A place where administrative, professional, or official work is carried out. | 3 | offices, office, office |
| **Outer space** | The physical universe beyond Earth’s atmosphere. | 3 | hyperspace, galaxy, extraterrestrial |
| **Rehabilitation** | The process of restoring health, ability, or normal life after illness, injury, or imprisonment. | 3 | rehabilitation, rehab centres for addicts |
| **Report** | A written or spoken account presenting information, findings, or analysis of an event or situation. | 3 | reporting, reports, reports on events/notifications |
| **Rights - negative** | lemma right - negative | 3 | too little right, no rights |
| **Tolerance** | The willingness to accept differences in opinions, beliefs, or behaviours. | 3 | tolerance, tolerance, tolerance for other denominations- preferences |
| **Whistleblowing** | The act of exposing wrongdoing, misconduct, or illegal activity within an organisation. | 3 | whistleblowing protection, whistleblowers (people who report wrongdoing), whistleblowing safeguards |
| **Benefits negative** | Adverse consequences or criticisms related to support systems, welfare, or advantages. | 2 | stop benefits, lowered benefits |
| **Conformity** | Behaviour that aligns with social norms, rules, or expectations. | 2 | certificate of conformity (corrected typo), conformity |
| **Consensus** | General agreement or shared opinion among a group. | 2 | consensus |
| **Crowd** | A large number of people gathered together, often in a public place. | 2 | crowding, crowd |
| **Despair** | Emotion | 2 | despair, desperate |
| **Enthusiasm** | Emotion | 2 | enthusiasm, overzealous |
| **Equality and diversity - negative** | Opposition or resistance to inclusion, fairness, or representation of different groups. | 2 | diversity needs tempering, transgender insanity |
| **Expansion** | The process of increasing in size, scope, or range. | 2 | expansion |
| **Expert** | A person with extensive knowledge or skill in a particular field. | 2 | expert, specialised |
| **Frustration** | The feeling of being upset or annoyed due to obstacles or unfulfilled expectations. | 2 | early frustration, frustration |
| **Gap** | A difference or disparity between groups, conditions, or levels. | 2 | gap, gaps |
| **Indulgence** | The act of yielding to one’s desires or allowing excessive leniency or pleasure. | 2 | indulgent, indulgent |
| **Luxury** | A state of great comfort, elegance, or material wealth beyond basic needs. | 2 | luxury, luxury |
| **Negative discourses or attitudes** | Expressions or viewpoints characterised by pessimism, hostility, or criticism. | 2 | badgering, protection from incitement and slander |
| **Pride** | Emotion | 2 | proud of my country, pride |
| **Research topics and positive evaluation** | research topics and positive evaluation. | 2 | protective policies are a good choice for study, behaviour studies |
| **Stalk** | To follow or pursue someone secretly and persistently. | 2 | stalking, stalkers |
| **Stress** | Mental or physical strain resulting from demanding or adverse circumstances. | 2 | stressful, stress |
| **Vaccination - no** | Opposition to or rejection of vaccines and immunisation practices. | 2 | no vacines, no vax (anti vacine) |
| **Affection** | Affection | 1 | affection |
| **Apologetic** | **sorry** | 1 | sorry |
| **Emotionless** | Absence of emotion. | 1 | without emotion |
| **Investment** | The allocation of money, time, or resources with the expectation of future benefit. | 1 | investment is needed |
| **Unsupported** | Lacking assistance, justification, or backing from others or evidence. | 1 | unsupported |

**Table S2**

*The central core of the SRs of protective policies and the emotional components held by participants from each of the 11 countries*

| Country | *Central Core* | *n* | MOE | Emotional Components | *n* | | MOE | | Quadrant |
| --- | --- | --- | --- | --- | --- | --- | --- | --- | --- |
| CZ | Police and security forces | 268 | 2.09 | **Fear** | **5** | | **3.80** | | **2P** |
| MOE = 2.60 | Military | 267 | 2.21 | Disgust | 2 | | 4.00 | | 2P |
| Inter. Freq.≥ 27 | Law and order | 174 | 2.48 | Sadness | 2 | | 3.50 | | 2P |
|  | Safety and security | 143 | 2.21 | Pride | 1 | | 2.00 | | 2P |
|  | Criticism | 79 | 2.44 | Satisfaction | 1 | | 2.00 | | 2P |
|  | Immigration | 75 | 2.32 |  |  | | |  |  |
|  | Foreign policy | 74 | 2.45 |  |  | | |  |  |
|  | COVID-19 | 52 | 2.35 |  |  | | |  |  |
|  | Defence | 48 | 2.29 |  |  | | |  |  |
|  | Border and territorial security | 41 | 2.02 |  |  | | |  |  |
|  | Insecurity | 41 | 1.66 |  |  | | |  |  |
|  | Positive | 36 | 2.36 |  |  | | |  |  |
|  | Limit | 33 | 2.52 |  |  | | |  |  |
|  | Welfare | 29 | 2.45 |  |  | | |  |  |
| DK | Police and security forces | 297 | 1.71 | **Calmness** | **8** | | | **3.50** | **2P** |
| MOE = 2.50 | Military | 166 | 2.02 | **Fear** | **5** | | | **2.00** | **Contrast** |
| Inter. Freq.≥ 27 | Crime | 92 | 2.49 | Dissatisfaction | 2 | | | 2.50 | 2P |
|  | Criticism | 92 | 2.29 | Love | 2 | | | 2.00 | Contrast |
|  | Foreign policy | 88 | 2.46 | Frustration | 1 | | | 5.00 | 2P |
|  | Defence | 87 | 1.98 | Gratitude | 1 | | | 2.00 | Contrast |
|  | Welfare | 81 | 2.17 |  |  | | |  |  |
|  | Safety and security | 79 | 2.29 |  | |  | |  |  |
|  | Surveillance and monitoring | 54 | 2.11 |  | |  | |  |  |
|  | War | 31 | 2.32 |  | |  | |  |  |
| FR | Police and security forces | 383 | 1.93 | **Fear** | | **8** | | **2.88** | **2P** |
| MOE = 2.50 | Criticism | 212 | 2.44 | Tranquillity | | 3 | | 3.33 | 2P |
| Inter. Freq.≥ 26 | Safety and security | 148 | 2.03 | Disappointment | | 2 | | 3.00 | 2P |
|  | Surveillance and monitoring | 108 | 2.39 | Anger | | 1 | | 4.00 | 2P |
|  | Military | 95 | 2.40 | Anxiety | | 1 | | 5.00 | 2P |
|  | Insecurity | 87 | 2.00 | Disgust | | 1 | | 3.00 | 2P |
|  | Healthcare and well-being | 63 | 2.27 | Dissatisfaction | | 1 | | 2.00 | Contrast |
|  | Immigration | 51 | 2.12 |  | |  | |  |  |
|  | Welfare | 49 | 1.94 |  | |  | |  |  |
|  | Terrorism | 37 | 2.03 |  | |  | |  |  |
|  | Child and childcare | 35 | 1.89 |  | |  | |  |  |
| DE | Police and security forces | 374 | 1.89 | **Fear** | | **7** | | **2.29** | **Contrast** |
| MOE = 2.50 | Law and order | 249 | 2.40 | Calmness | | 2 | | 4.00 | 2P |
| Inter. Freq.≥ 28 | Military | 121 | 2.26 | Disappointment | | 1 | | 4.00 | 2P |
|  | Safety and security | 99 | 2.40 | Hate | | 1 | | 2.00 | Contrast |
|  | Border and territorial security | 98 | 2.27 | Hope | | 1 | | 1.00 | Contrast |
|  | Immigration | 78 | 1.86 |  | |  | |  |  |
|  | Criticism | 65 | 2.42 |  | |  | |  |  |
|  | Crime | 48 | 2.38 |  | |  | |  |  |
|  | Welfare | 45 | 2.11 |  | |  | |  |  |
|  | Asylum and refugee | 44 | 1.82 |  | |  | |  |  |
|  | Democracy | 35 | 2.31 |  | |  | |  |  |
|  | Barriers | 29 | 2.10 |  | |  | |  |  |
| IE | Law and order | 369 | 2.50 | **Joy** | | **16** | | **2.81** | **2P** |
| MOE = 2.60 | Safety and security | 311 | 2.46 | **Fear** | | **8** | | **2.63** | **2P** |
| Inter. Freq.≥ 32 | Police and security forces | 253 | 2.23 | **Calmness** | | **5** | | **3.60** | **2P** |
|  | Criticism | 164 | 2.35 | Disappointment | | 4 | | 3.00 | 2P |
|  | Welfare | 123 | 2.42 | Anxiety | | 3 | | 4.33 | 2P |
|  | Child and childcare | 119 | 2.49 | Hope | | 2 | | 4.50 | 2P |
|  | Insurance | 87 | 2.00 | Love | | 2 | | 3.00 | 2P |
|  | Housing and rent | 71 | 2.45 | Disgust | | 2 | | 5.00 | 2P |
|  | Human rights | 70 | 2.29 | Anger | | 1 | | 1.00 | Contrast |
|  | Guards and safeguarding | 67 | 2.12 | Gratitude | | 1 | | 3.00 | 2P |
|  | Positive | 63 | 2.49 | Hate | | 1 | | 4.00 | 2P |
|  | Insecurity | 60 | 2.13 | Pride | | 1 | | 3.00 | 2P |
|  | Immigration | 44 | 2.41 | Shame | | 1 | | 4.00 | 2P |
|  | Data | 42 | 2.02 | Tranquillity | | 1 | | 2.00 | 2P |
|  | Probability and doubt | 39 | 1.69 |  | |  | |  |  |
| IL | Military | 476 | 1.98 | **Fear** | | **48** | | **3.15** | **2P** |
| MOE = 2.70 | Police and security forces | 277 | 2.51 | **Calmness** | | **9** | | **3.11** | **2P** |
| Inter. Freq.≥ 29 | Criticism | 200 | 2.52 | **Disappointment** | | **9** | | **2.89** | **2P** |
|  | Law and order | 166 | 2.61 | **Shame** | | **7** | | **2.43** | **Contrast** |
|  | Safety and security | 130 | 2.52 | Love | | 4 | | 4.25 | 2P |
|  | Insecurity | 102 | 2.11 | Anxiety | | 3 | | 4.33 | 2P |
|  | Air security and defence | 86 | 2.41 | Joy | | 2 | | 3.00 | 2P |
|  | Confidence | 78 | 2.23 | Sadness | | 2 | | 2.50 | Contrast |
|  | War | 52 | 2.54 | Disgust | | 2 | | 2.00 | Contrast |
|  | Negligence and abandonment | 37 | 2.11 | Guilt | | 2 | | 2.50 | Contrast |
|  | Positive | 35 | 2.43 | Anger | | 1 | | 4.00 | 2P |
|  |  |  |  | Despair | | 1 | | 3.00 | 2P |
|  |  |  |  | Dissatisfaction | | 1 | | 2.00 | Contrast |
|  |  |  |  | Frustration | | 1 | | 5.00 | 2P |
|  |  |  |  | Hate | | 1 | | 3.00 | 2P |
| IT | Police and security forces | 225 | 2.12 | **Calmness** | | **9** | | **3.44** | **2P** |
| MOE = 2.60 | Safety and security | 211 | 2.06 | **Fear** | | **8** | | **2.75** | **2P** |
| Inter. Freq.≥ 27 | Law and order | 125 | 2.40 | **Disgust** | | **6** | | **1.67** | **Contrast** |
|  | Healthcare and well-being | 87 | 2.51 | Joy | | 3 | | 4.00 | 2P |
|  | Insecurity | 87 | 1.89 | Love | | 3 | | 4.33 | 2P |
|  | Welfare | 63 | 2.13 | Sadness | | 3 | | 2.33 | Contrast |
|  | Immigration | 61 | 2.48 | Hope | | 2 | | 2.00 | Contrast |
|  | Military | 51 | 1.82 | Anxiety | | 2 | | 4.50 | 2P |
|  | Help and support | 48 | 2.56 | Anger | | 1 | | 5.00 | 2P |
|  | Emergency and rescue | 35 | 2.29 | Disappointment | | 1 | | 3.00 | 2P |
|  | Control and order | 33 | 2.49 | Hate | | 1 | | 4.00 | 2P |
|  |  |  |  | Tranquillity | | 1 | | 5.00 | 2P |
| PL | Safety and security | 246 | 2.04 | **Calmness** | | **34** | | **2.79** | **2P** |
| MOE = 2.50 | Criticism | 152 | 2.30 | **Fear** | | **10** | | **2.00** | **2P** |
| Inter. Freq.≥ 25 | Healthcare and well-being | 144 | 2.21 | **Joy** | | **6** | | **3.00** | **2P** |
|  | Military | 128 | 2.31 | Satisfaction | | 3 | | 4.00 | 2P |
|  | Police and security forces | 103 | 2.39 | Anxiety | | 2 | | 2.50 | Contrast |
|  | Politics | 92 | 2.15 | Despair | | 1 | | 2.00 | Contrast |
|  | Border and territorial security | 85 | 2.31 | Disappointment | | 1 | | 1.00 | Contrast |
|  | Insecurity | 77 | 1.92 | Disgust | | 1 | | 1.00 | Contrast |
|  | Welfare | 61 | 2.36 | Dissatisfaction | | 1 | | 1.00 | Contrast |
|  | Crime | 55 | 2.40 | Enthusiasm | | 1 | | 1.00 | Contrast |
|  | Data | 37 | 2.22 | Guilt | | 1 | | 2.00 | Contrast |
|  | Positive | 32 | 2.13 | Sadness | | 1 | | 3.00 | 2P |
| PT | Police and security forces | 406 | 1.81 | **Fear** | | **15** | | **3.07** | **2P** |
| MOE = 2.60 | Safety and security | 302 | 2.22 | **Tranquillity** | | **11** | | **2.64** | **2P** |
| Inter. Freq.≥ 33 | Criticism | 183 | 2.49 | Calmness | | 3 | | 1.67 | Contrast |
|  | Healthcare and well-being | 180 | 2.49 | Love | | 2 | | 4.50 | 2P |
|  | Welfare | 107 | 2.24 | Shame | | 2 | | 2.00 | Contrast |
|  | Crime | 82 | 2.56 | Anxiety | | 1 | | 3.00 | 2P |
|  | Insecurity | 76 | 2.18 | Dissatisfaction | | 1 | | 2.00 | Contrast |
|  | Control and order | 43 | 2.23 | Hate | | 1 | | 3.00 | 2P |
|  | Data | 36 | 2.00 |  | |  | |  |  |
|  | Human rights | 33 | 2.24 |  | |  | |  |  |
| SE | Police and security forces | 328 | 1.88 | **Fear** | | **7** | | **3.29** | **2P** |
| MOE = 2.60 | Criticism | 179 | 2.15 | Calmness | | 3 | | 1.67 | Contrast |
| Inter. Freq.≥ 30 | Law and order | 144 | 2.59 | Hate | | 3 | | 4.33 | 2P |
|  | Military | 142 | 2.37 | Anxiety | | 2 | | 5.00 | 2P |
|  | Crime | 102 | 2.44 | Hope | | 2 | | 3.50 | 2P |
|  | Politics | 88 | 2.36 | Shame | | 2 | | 2.50 | Contrast |
|  | Defence | 83 | 2.19 | Disappointment | | 1 | | 5.00 | 2P |
|  | Safety and security | 71 | 2.09 | Gratitude | | 1 | | 2.00 | Contrast |
|  | Economy and finances | 63 | 2.44 | Joy | | 1 | | 5.00 | 2P |
|  | Insecurity | 58 | 2.05 |  | |  | |  |  |
|  | Surveillance and monitoring | 48 | 2.48 |  | |  | |  |  |
|  | Positive | 46 | 1.80 |  | |  | |  |  |
|  | Immigration | 39 | 2.46 |  | |  | |  |  |
|  | Probability and doubt | 38 | 2.37 |  | |  | |  |  |
|  | Insurance | 34 | 2.21 |  | |  | |  |  |
|  | Equality, diversity, and inclusion | 33 | 2.58 |  | |  | |  |  |
| UK | Law and order | 272 | 2.44 | **Calmness** | | **6** | | **3.33** | **2P** |
| MOE = 2.60 | Safety and security | 262 | 2.28 | **Joy** | | **6** | | **3.33** | **2P** |
| Inter. Freq.≥ 28 | Police and security forces | 226 | 1.81 | Hope | | 3 | | 2.67 | 2P |
|  | Healthcare and well-being | 122 | 2.39 | Enthusiasm | | 2 | | 5.00 | 2P |
|  | Criticism | 109 | 2.30 | Fear | | 2 | | 3.00 | 2P |
|  | Equality, diversity, and inclusion | 83 | 2.57 | Love | | 1 | | 4.00 | 2P |
|  | Human rights | 81 | 2.58 | Tranquillity | | 1 | | 1.00 | Contrast |
|  | Welfare | 74 | 2.60 |  | |  | |  |  |
|  | Guards and safeguarding | 67 | 2.31 |  | |  | |  |  |
|  | Insurance | 54 | 2.06 |  | |  | |  |  |
|  | Data | 41 | 2.32 |  | |  | |  |  |
|  | Defence | 33 | 2.12 |  | |  | |  |  |
|  | Immigration | 28 | 2.46 |  | |  | |  |  |

**Figure S1**

*Similarity (co-occurrence) network of emotional components and other categories in the social representations of protective policies in the overall results.*

**Figure S2**

*Similarity (co-occurrence) network of emotional components and other categories in the social representations of protective policies in the Czech Republic.*

**Figure S3**

*Similarity (co-occurrence) network of emotional components and other categories in the social representations of protective policies in Denmark.*

**Figure S4**

*Similarity (co-occurrence) network of emotional components and other categories in the social representations of protective policies in France.*

**Figure S5**

*Similarity (co-occurrence) network of emotional components and other categories in the social representations of protective policies in Germany.*

**Figure S6**

*Similarity (co-occurrence) network of emotional components and other categories in the social representations of protective policies in Ireland.*

**Figure S7**

*Similarity (co-occurrence) network of emotional components and other categories in the social representations of protective policies in Israel.*

**Figure S8**

*Similarity (co-occurrence) network of emotional components and other categories in the social representations of protective policies in Italy.*

**Figure S9**

*Similarity (co-occurrence) network of emotional components and other categories in the social representations of protective policies in Poland.*

**Figure S10**

*Similarity (co-occurrence) network of emotional components and other categories in the social representations of protective policies in Portugal.*

**Figure S11**

*Similarity (co-occurrence) network of emotional components and other categories in the social representations of protective policies in Sweden.*

**Figure S12**

*Similarity (co-occurrence) network of emotional components and other categories in the social representations of protective policies in the United Kingdom.*
